# *Helicobacter pylori* contributes to GC progression, possibly via the MSTRG.10627.1/miR-142-5p/ADAMTS5 pathway

**DOI:** 10.3389/fmicb.2025.1686246

**Published:** 2026-02-02

**Authors:** Zhipeng Yin, Jianwei Xiang, Pengbo Guo, Nianli Zhang, Xiaoqian Lv, Yu Wang, Yi Wu, Nianhua Zhang, Gang Lv, Yinghui Zhao

**Affiliations:** 1 Laboratory of Metabolism and Gastrointestinal Tumor, The First Affiliated Hospital of Shandong First Medical University, Jinan, Shandong, China; 2 Department of Pathogen Biology, School of Clinical and Basic Medical Sciences, Shandong First Medical University & Shandong Academy of Medical Sciences, Jinan, Shandong, China; 3 Department of Radiology, Shandong Provincial Hospital, Cheeloo College of Medicine, Shandong University, Jinan, Shandong, China; 4 Department of Anesthesiology, Weifang People’s Hospital, Weifang, Shandong, China

**Keywords:** ADAMTS5, GC, *H. pylori*, miR-142-5p, MSTRG.10627.1

## Abstract

**Background:**

*Helicobacter pylori* (*H. pylori*) is a class I carcinogen that induces gastric cancer (GC). The mechanisms underlying its induction of GC are not fully understood. The role of the MSTRG.10627.1/miR-142-5p/ADAMTS5 pathway induced by *H. pylori* in GC was investigated in this study.

**Methods:**

RNA sequencing and bioinformatics analysis were used to screen a regulatory pathway lncRNA-miRNA-mRNA. The dual luciferase reporter was used to evaluate interactions between the lncRNA and the miRNA or between the miRNA and the mRNA. The cellular biological effects of ADAMTS5 were detected using clonogenic formation and cell migration assays. A western blot was used to assess the protein expression of the pathway. The role of ADAMTS5 in tumor growth and metastasis was validated in nude mice using subcutaneous and tail vein injections.

**Results:**

A regulatory axis lncRNA (MSTRG.10627.1)-miRNA (miR-142-5p)-mRNA (ADAMTS5) with the highest correlation coefficient was screened out. The combinations between MSTRG.10627.1 and miR-142-5p or between miR-142-5p and ADAMTS5 were verified. The expression of ADAMTS5 was down-regulated by *H. pylori* (*p* < 0.05). The proliferation, migration, and invasion of GC cells were increased by down-regulation of ADAMTS5 (*p* < 0.05); however, the proliferation, migration, and invasion of GC cells were inhibited by the overexpression of ADAMTS5 (*p* < 0.05). After silencing of ADAMTS5 in GC cell lines, western blot showed that the expression of PI3K protein and the phosphorylation level of AKT protein were increased (*p* < 0.05), and the expression of tumor suppressor p53 was inhibited (*p* < 0.05). However, overexpression of ADAMTS5 in GC cells induced the opposite results (*p* < 0.05). The results of the subcutaneous tumor model in nude mice showed that tumor weight and volume increased after silencing of ADAMTS5 (*p* < 0.05). The metastasis of GC cells in the metastatic tumor model was inhibited by overexpression of ADAMTS5 (*p* < 0.05).

**Conclusion:**

The expression of ADAMTS5 was down-regulated by *H. pylori* through regulating a probable pathway (MSTRG.10627.1-miR-142-5p-ADAMTS5). Moreover, down-regulated ADAMTS5 induced PI3K protein, up-regulated phosphorylated AKT protein, and down-regulated p53, which plays an important role in the induction of GC.

## Introduction

The prevalence of *Helicobacter pylori* (*H. pylori*) worldwide has exceeded 50% (Sven et al., 2024). *H. pylori* infection is a major public health problem worldwide. It significantly increases the risk of chronic gastritis, gastroesophageal reflux disease, functional dyspepsia, gastric ulcer, gastric cancer (GC), etc. (Hatta et al., 2021). Although the overall prevalence of *H. pylori* has declined, the incidence and mortality rates associated with GC are still high.

Although GC was a multifactorial disease, *H. pylori* was one of the main risk factors for the occurrence and development. Research has shown that the eradication of *H. pylori* significantly reduced the risk of first-degree relatives with a family history of GC (Choi et al., 2020). Most GC patients diagnosed with an advanced stage are prone to recurrence, have a low 5-year survival rate, and have a very poor prognosis (Tan, 2019). Considering the above, finding early diagnosis markers and treatment targets is an important way to reduce harm.

Generally, non-coding RNA (ncRNA) does not code for proteins and is divided into short non-coding RNA (<30 nts) and long non-coding RNA (>200 nts) based on length (Tani et al., 2019). Long non-coding RNA mainly includes long-chain non-coding RNA (lncRNA) and circular RNA (circRNA). Recent studies have shown that lncRNAs not only participate in various physiological and pathological processes but also in the regulation of malignant tumors (Calin and Croce, 2009; Sur and Ray, 2022). lncRNA can interact with miRNA via the competitive endogenous RNA (ceRNA) to regulate mRNA expression (Zhou et al., 2019). lncRNA has been shown to act as a ceRNA and significantly impact tumor pathogenesis. lncRNAs in the cytoplasm or nucleus participate in the regulation of gene expression in various ways, which may also be a predictor of poor prognosis of cancer (Chen et al., 2022). The role of lncRNA in the development and progression of GC remains poorly understood.

Metalloproteinases (MPs) are a large family of proteases with metal ions in their active centers. According to different domains, MPs were divided into various subtypes, mainly including matrix metalloproteinases (MMPs), integrin metalloproteinases (ADAMs), and ADAMs with thrombospondin motifs (ADAMTS) (Saw et al., 2019), which had multiple functions such as protein hydrolysis, cell adhesion, and extracellular matrix remodeling (Klein et al., 2018). MPs’ family members were overexpressed in various cancers, including colorectal cancer (Schwegmann et al., 2016), lung cancer (Gong et al., 2016), breast cancer (Jiang and Li, 2021), ovarian cancer (Carey et al., 2021), and GC (Chang et al., 2014), etc. It participated in the occurrence, development, invasion, metastasis, and other pathological processes of tumors by regulating signal transduction and tumor microenvironment (Sun et al., 2015). ADAMTS5 is a member of the metalloproteinase family containing type I platelet-responsive protein and integrin. ADAMTS5 plays different roles in different tumors, and its molecular mechanisms vary accordingly (Kelwick et al., 2015). Research showed that ADAMTS5 inhibited the growth of mouse melanoma through an anti-angiogenesis mechanism (Renganathan et al., 2018). Another research found that it acted as a tumor suppressor by inhibiting migration, invasion, and angiogenesis in human GC in cell lines and tissues (Huang et al., 2019). In non-small cell lung cancer, ADAMTS5 played a role in promoting cancer by degrading the extracellular matrix to increase the migration and invasion ability of the cancer (Gu et al., 2016). However, the role and regulatory mechanism of ADAMTS5 in GC remain unclear.

In this study, we found that ADAMTS5 was differentially expressed across GC cell lines using full-length transcriptional sequencing technology. A ceRNA regulatory network of lncRNA-miRNA-mRNA was constructed using RNA sequencing and online bioinformatics analysis. The ADAMTS5 expression in GC cell lines was decreased after infection with *H. pylori*. Overall, in this study, we primarily investigated the regulation of GC by *H. pylori* through the tumor suppressor molecule ADAMTS5 and the ceRNA regulatory axis, aiming to identify new candidate diagnostic markers and therapeutic targets for GC.

## Materials and methods

### Cell lines, bacterial strain, plasmids, and siRNA

The human GC cell lines BGC-823, AGS, MKN-28, MKN-45, MGC-803, and NCI-N87 were purchased from Wuhan Purosa Life Technology Co., Ltd. The *H. pylori* 26,695 strain was donated by Jihui Jia Research Group of Shandong University.

miR-142-5p mimics, miR-142-5p inhibitor, ADAMTS5 siRNA, ADAMTS5 eukaryotic expression vector, MSTRG.10627.1 siRNA, and MSTRG.10627.1 eukaryotic expression vector were purchased from Guangzhou Ruibo Biotechnology Co., Ltd. The sequences were listed in Supplementary Table S1. PI3K/AKT inhibitor Miltefosine was purchased from Shanghai Lianzu Biotechnology Co., Ltd.

### Cell transfection

BGC-823, AGS, and MKN-28 were inoculated into a six-well plate with 1 × 10^6^/well. 2 mL of culture medium was added to each well, then the plate was placed in a cell incubator. When the cell density reached 75%, it could be used for subsequent transfection. 2 μg plasmid/small interference/miRNA mimic/inhibitor was diluted to 125 μL by Opti-MEM, which was marked as solution A. 3.75 μL Lipofectamine^™^ 3000 was diluted to 125 μL by Opti-MEM, which was marked as solution B. Solutions A and B were fully mixed and kept at 20 °C for 15 min. The original culture medium in the 6-well plate was removed, and 250 μL of the corresponding mixture was added to each well. After that, the fresh medium without the double antibody was added to each well, bringing the total volume to 2 mL. The transfected cells were incubated in a 37 °C, 5% CO_2_ incubator for 2 days.

### Cell biological activity test

BGC-823, AGS, and MKN-28 were inoculated in a 96-well plate with 1 × 10^4^ cells/well. AGS and MKN-28 were transfected with the ADAMTS5 eukaryotic expression vector, while the cells transfected using the empty vector were recognized as controls. BGC-823 was transfected with ADAMTS5 siRNA, while the cells transfected with unrelated sequences were identified as controls. After 24 h of transfection, the previous medium was removed, and 100 μL of fresh medium (without fetal bovine serum) and 10 μL of CCK-8 reagent were added. After that, the cells were incubated in a 37 °C, 5% CO_2_ incubator for 1 h, and then the absorbance was measured at 450 nm using an enzyme marker.

After 24 h of transfection, the clonogenic ability of the cells was tested by a clonogenic assay. The transfected cells were digested with trypsin and resuspended to form a single-cell suspension. The cell concentration was adjusted to 250 cells/mL, and 2 mL of diluted single-cell suspension was added to the corresponding hole in the 6-well plate. The cells were incubated in a 37 °C, 5% CO_2_ incubator for 10 days. When cell colony formation was observed at the bottom of the well, the medium was removed, and the cells were washed three times with sterile phosphate-buffered saline (PBS). After that, the cells were fixed for 20 min with 4% paraformaldehyde and then washed three times with sterile PBS. The wells were added to 2 mL of Giemsa solution for 20 min at 20 °C. Finally, the cells were photographed and counted after being washed three times using sterile PBS.

The migratory ability of GC cells with ADAMTS5 overexpression and with silent expression was assessed using the wound healing assay. The cells were inoculated in a 6-well plate (1 × 10^6^ cells/well) and cultured in a 5% CO_2_ cell incubator at 37 °C. When the cells fully covered the bottom of the six-well plate, the bottom was scratched. The wells were slowly rinsed using PBS to remove cellular debris. After that, the cells were placed in a culture medium without fresh serum and cultured in a 5% CO_2_ cell incubator at 37 °C for 0, 24, and 48 h. Finally, the cells were photographed using an inverted microscope (CKX53, OLYMPUS, Japan), and the length and width of the scratch were calculated.

The invasive ability of the cells was detected using a transwell assay 24 h after transfection. After being melted overnight at 4 °C, the Matrigel glue was diluted with serum-free medium at a ratio of 1:5. The upper chamber was filled with 45 μL of the front mixture and incubated in a cell incubator for 5 h. Then, the upper chamber was added with 100 μL of single-cell suspension (containing 5 × 10^4^ cells), while 700 μL of complete medium containing 20% fetal bovine serum was added into the lower chamber. The transwell chamber was incubated in a cell incubator for 24 h. The culture medium in the upper chamber was removed. After washing with sterile PBS three times, the cells were fixed using 100 μL 4% formaldehyde for 20 min. After removing the formaldehyde, the upper chamber was washed three times with sterile PBS. Then, 100 μL 0.1% crystal violet was used to stain the cells for 20 min. After removing the crystal violet, the upper chamber was washed with sterile PBS three times. A medical cotton swab was used to wipe the cells in the upper chamber, and the cells that passed through it were counted using an inverted microscope from five random fields of view. MKN-28 cells with ADAMTS5 overexpression and silent expression were treated with 10 μM PI3K/AKT inhibitor miltefosine, and the protein was extracted for the following assay.

### Cell infected with *Helicobacter pylori*

BGC-823 cells were inoculated onto a six-well plate (5 × 10^5^ cells/well) and placed in a 37 °C, 5% CO_2_ cell incubator for 2 days. *H. pylori* were collected and added into a six-well plate at multiplicity of infection (MOI) = 100:1, while the control group was added an equal amount of serum-free and antibiotic-free medium. The bacteria and cells were cultured together for 1, 2, 3, 6, 12, and 24 h, respectively, and then the total RNA and protein of the cells were extracted.

### RNA sequencing experiment

AGS, MKN-28, NCI-N87, BGC-823, MKN-45, and MGC-803 were inoculated in 6-well plates. When the cell density reached 90%, the total RNA was extracted. Then, the RNA was sequenced by Parsonor Biotechnology Company in Shanghai, China, based on the Illumina HiSeq sequencing platform (Kukurba and Montgomery, 2015). Based on different types of whole genome annotation information, grouping testing and characteristic sequence analysis were performed. The DESeq2 package in the R language was used to perform principal component analysis on the samples to evaluate expression differences among samples and assess separation between groups. The transcripts with class codes “u,” “I,” and “x,” corresponding to intergenic regions, intron regions, or antisense strand long non-coding RNA (lncRNA), had been collected. lncRNA with coverage ≥3 in at least one sample was selected for expression level analysis. Three tools, CNCI, PLEK, and Pfam, were used to predict coding potential. The transcript was retained when identified as “non-coding” by at least two of these tools. The expression of relevant genes was statistically analyzed, and the differences in expression were analyzed. Based on the lncRNA, miRNA, and mRNA expressions of different genes, miRanda was used to predict the relationship between the two target gene sequences (mRNA and lncRNA). Based on miRNA expression, a regulatory network of lncRNA-miRNA-mRNA was constructed. For an lncRNA-miRNA-mRNA with a ceRNA pairing relationship (screening results with a sensitive correlation >0.3), the mRNA (target genes) was analyzed using GO and KEGG enrichment analyses. (For detailed information, please refer to the attachment of the Transcriptome lncRNA report).

### Quantitative reverse transcription PCR

Total RNA of cells and tumor tissue was extracted using the previously described method (Kukurba and Montgomery, 2015). The reverse transcription system of mRNA and lncRNA consisted of 4 μL dNTP Mix (2.5 mM each), 2 μL primer mix, 1 μg RNA template, 4 μL 5 × RT buffer, 2 μL DTT (0.1 M), 1 μL HiFiScript (200 U/uL), and an appropriate amount of RNase-free water (total volume of system is 20 μL). Reverse transcription was performed with the Prime-Script^™^ RT reagent kit (Takara Bio, Inc.) and in an ABI 7000 (Applied Biosystems; Thermo Fisher Scientific, Inc.) using the following cycles: 42 °C for 15 min and 85 °C for 5 min. The reverse transcription system of miRNA consisted of 2 μg total RNA, 1 μL 2 × miRNA RT Buffer, 2 μL miRNA RT enzyme mix, and an appropriate amount of RNase-free water (total volume of system is 10 μL). Reverse transcription was performed with the Prime-Script^™^ RT reagent kit (Takara Bio, Inc.) and in an ABI 7000 (Applied Biosystems; Thermo Fisher Scientific, Inc.) using the following cycles: 42 °C for 60 min and 95 °C for 3 min.

The PCR reaction system of mRNA and lncRNA consisted of 10 μL 2 × magic SYBR mixture, 1 μL forward primer (10 μM), 1 μL reverse primer (10 μM), 2 μL cDNA, and RNase-free water (total volume of system is 20 μL). The PCR was performed with the Prime-Script^™^ RT reagent kit (Takara Bio, Inc.) on an ABI 7000 (Applied Biosystems; Thermo Fisher Scientific, Inc.) using the following thermocycling cycles: 95 °C for 30 s; 60 °C for 30 s for 40 cycles; and 60 °C for 30 s. The experiments were performed in triplicate, and expression was calculated with the 2^−ΔΔCq^ quantification method. The PCR reaction system of miRNA consisted of 10 μL 2 × miRcute plus miRNA PreMix, 0.4 μL forward primer, 0.4 μL reverse primer (10 μM), 2 μL cDNA, and an appropriate amount of RNase-free water (total volume of system is 20 μL). PCR was performed with the Prime-Script^™^ RT reagent kit (Takara Bio, Inc.) and in an ABI 7000 (Applied Biosystems; Thermo Fisher Scientific, Inc.) using the following thermocycling cycles: 95 °C for 15 min for 1 cycle, 63 °C for 30 s for 5 cycles, 94 °C for 20 s for 40 cycles, and 60 °C for 34 s. The experiments were performed in triplicate, and the expression level was calculated with the 2^−ΔΔCq^ quantification method. The primers were listed in Supplementary Table S2.

### Western blot

The total protein of cells and tumor tissue was extracted using the previously described method (Ma et al., 2020). The protein was quantified using a bicinchoninic acid protein assay kit, mixed using 50 μL of SDS-PAGE sample buffer, then boiled for 5 min. After that, 5 μg per lane was loaded onto the polyacrylamide gel, and SDS-PAGE was performed with a 10% separating gel at 110 V for 60 min. The proteins were transferred onto polyvinylidene fluoride membranes (Beyotime Institute of Biotechnology) via electrophoresis at 300 mA for 60 min, using a Bio-Rad transfer system (Bio-Rad Laboratories, Inc.). The membranes were blocked for 8 h with skimmed milk (Beyotime Institute of Biotechnology) at 4 °C and probed with the corresponding antibody (rabbit) diluted in blocking buffer at an appropriate dilution (1:250, anti-ADAMTS5, Cat. No. ab41037; 1:250, anti-p53, Cat. No. ab131442; 1:1,000, anti-PI3K, Cat. No. C73F8; 1:1,000, anti-AKT, Cat. No. C67E7; 1:2,000, anti-pAKT, Cat. No. Ser473; 1:5,000, anti-GAPDH, Cat. No. 10494-1-AP) at 4 °C for 12 h. The membrane was then incubated for 2 h at 20 °C with horseradish peroxidase-labeled goat anti-rabbit IgG antibody (Cat. No. 18772; Millipore Sigma), diluted in blocking buffer at 1:10,000, and signals were detected using a chemiluminescent developer (Beyotime Institute of Biotechnology).

### Luciferase activity analysis

AGS and 293T cell lines were cultivated in Dulbecco’s modified Eagle’s medium (DMEM) with antibiotics and fetal bovine serum at 37 °C in humidified 5% CO_2_. When the cell fusion reached 60%, a transfection experiment was performed. To verify whether MSTRG.10627.1 can directly bind to miR-142-5p, the wild-type sequence (WT) and mutant sequence (Mut) of MSTRG.10627.1, as well as the miR-142-5p mimic and negative control (mimic NC), were constructed. Similarly, to verify whether there was a direct binding between miR-142-5p and the downstream target gene ADAMTS5, the wild-type (WT) and mutant (Mut) sequences of ADAMTS5, as well as the miR-142-5p mimic and negative control (mimic NC), were also constructed. The constructed vectors were transfected into AGS and 293T cell lines, respectively. Then their luciferase activity in the cells was measured using a dual-luciferase reporter assay system (Promega Corporation).

### Fluorescence *in situ* hybridization

To study the role of lncRNA MSTRG.10627.1, the online prediction website lncLocator^1^ was used, and fluorescence *in situ* hybridization (FISH) was used to detect its subcellular localization in the AGS cell line. The cells were cultivated in DMEM with antibiotics and fetal bovine serum in a 24-well plate at 37 °C, 5% CO_2_. When cell fusion reached 70%, hybridization was carried out. It was washed three times using PBS and fixed with 4% paraformaldehyde for 10 min at 20 °C. The well was filled with 1 mL of a cell-permeable solution and incubated for 5 min at 4 °C. The cell-permeable solution was removed, and then the cells were washed three times with PBS. lncRNA probe mix storage solution, U6, and 18S internal reference storage solution were mixed with 100 μL hybrid solution separately. The mixture was added into the wells (100 μL/well), and the cells were incubated in an incubator at 37 °C for 16 h. The cells were washed using SSC after removing the hybrid solution. After washing for 5 min with PBS, the cells were stained using DAPI for 10 min. Then, the cells were washed three times with PBS and placed on a slide. Finally, a drop of sealing agent was used to seal the slide, and a confocal microscope was used to detect it.

### Tumor proliferation and metastasis experiment in nude mice

BALB/c nude mice were purchased from Beijing Vital River Laboratory Animal Technology Co., Ltd., Animal License: SCXK (Jing) 2021-0006, and all animal experiments were approved by the Experimental Animal Ethics Committee of Shandong First Medical University & Shandong Academy of Medical Sciences (Ethical Approval Number, W2021030086). The nude mice were raised in an SPF animal laboratory for 1 week and then randomly divided into a blank control (Control) group, a negative control (si-NC) group, and an experimental (si-ADAMTS5) group. The right underarms of the experimental group and the negative control group of nude mice were injected subcutaneously with 200 μL BGC-823 cell suspension (cell density: 5 × 10^7^/mL). After subcutaneous tumorigenesis, the experimental group of nude mice was injected with the siRNA of ADAMTS5 (each nude mouse was injected with 20 μg), while the negative control group of nude mice was injected with a corresponding unrelated sequence RNA (each nude mouse was injected with 20 μg). All the mice were injected four times a week, with an interval of 2 days (each time 100 μL). The body weight and tumor volume in nude mice were measured and recorded. When there was a significant difference in the tumor volume between the experimental group and the control group, the nude mice were killed using cervical dislocation after anesthesia, and the subcutaneous tumor was collected. The tumor was weighed, and its length and diameter were measured to calculate the tumor volume.

BALB/c nude mice were raised as above, and then they were randomly divided into a blank control (Control) group, a negative control (NC) group, and an experimental (ADAMTS5) group. The experimental group mice were injected with 200 μL of ADAMTS5-overexpressing AGS cells via the caudal vein. The negative control group mice were injected with 200 μL normal AGS cells, while the blank control group was injected with 200 μL PBS using the same method. All the mice were injected at 2-day intervals. After 40 days, the nude mice were killed by cervical dislocation after anesthesia, and the lungs were collected to count the number of lung metastases.

### Statistical analysis

All experiments were performed three times. Values are expressed as the mean ± standard deviation. Statistically significant differences between the two groups were determined using the SNK method. ANOVA and the least-significant difference test were used for comparison among multiple groups using SPSS 22.0. The results of the western blot were analyzed using ImageJ software (v1.51; National Institutes of Health). A *p* < 0.05 was considered statistically significant.

## Results

### The lncRNA-miRNA-mRNA regulatory network of GC cell lines

**Figure 1:**
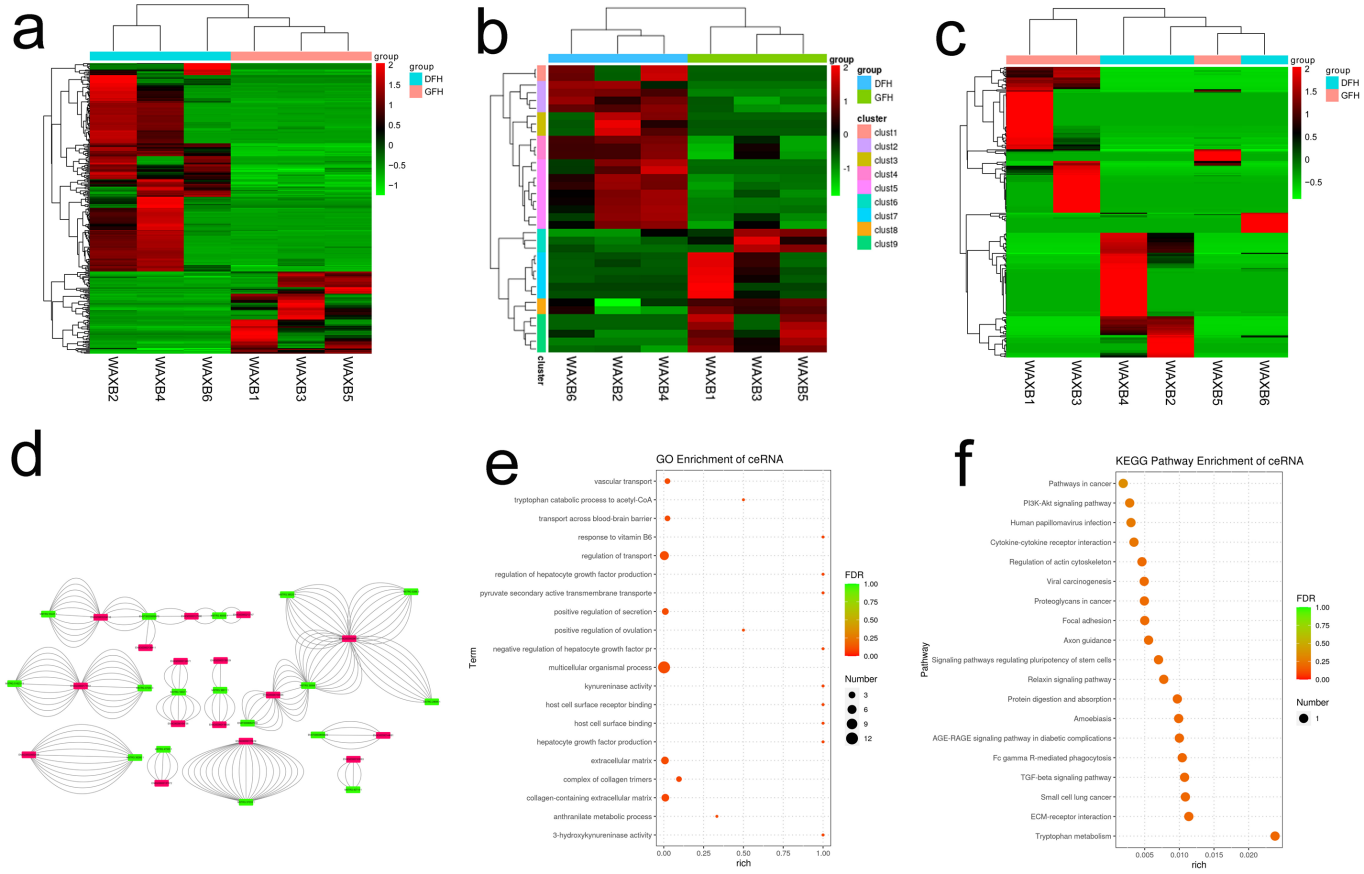
The heat maps and regulatory networks of lncRNA, miRNA, and mRNA were differentially expressed across six GC cells. **(a)** Heat map of mRNA. **(b)** Heat map of miRNA. **(c)** Heat map of lncRNA. **(d)** lncRNA-miRNA-mRNA regulatory network of GC. Green indicates lncRNA, red indicates mRNA, and the middle line indicates miRNA. **(e)** GO enrichment analysis of lncRNA-miRNA-mRNA regulatory network in GC. **(f)** KEGG enrichment analysis of lncRNA-miRNA-mRNA regulatory network in GC.

The mRNA levels between AGS, NCIA-87, MKN-28 and MGC-803, MKN-45, and BGC-823 presented significant differences (Figure 1a, *p* < 0.05). There was a significant difference in miRNA between AGS, NCIA-87, MKN-28, and MGC-803, MKN-45, and BGC-823 (Figure 1b, *p* < 0.05). The lncRNA levels showed differences among the six cell lines (Figure 1c, *p* < 0.05). An lncRNA-miRNA-mRNA regulatory network was constructed with an optimized process. An interactive regulatory network consisting of 17 lncRNAs, 32 miRNAs, and 16 mRNAs was obtained (Supplementary Table S2). The lncRNA-miRNA-mRNA regulatory network of GC was shown in Figure 1d. GO and KEGG signal pathway analyses of the target genes of the GC-related lncRNA-miRNA-mRNA regulatory network were performed. In the functional analysis of GO, the main enrichment levels were multicellular biological processes, regulatory transport, extracellular matrix, and extracellular matrix containing collagen (Figure 1e). KEGG pathway analysis, as shown in Figure 1f, primarily involved the PI3K-AKT, relaxin, and TGF-*β* signaling pathways.

### *Helicobacter pylori* changed the levels of ADAMTS5, MSTRG.10627.1, and miR-142-5p

**Figure 2:**
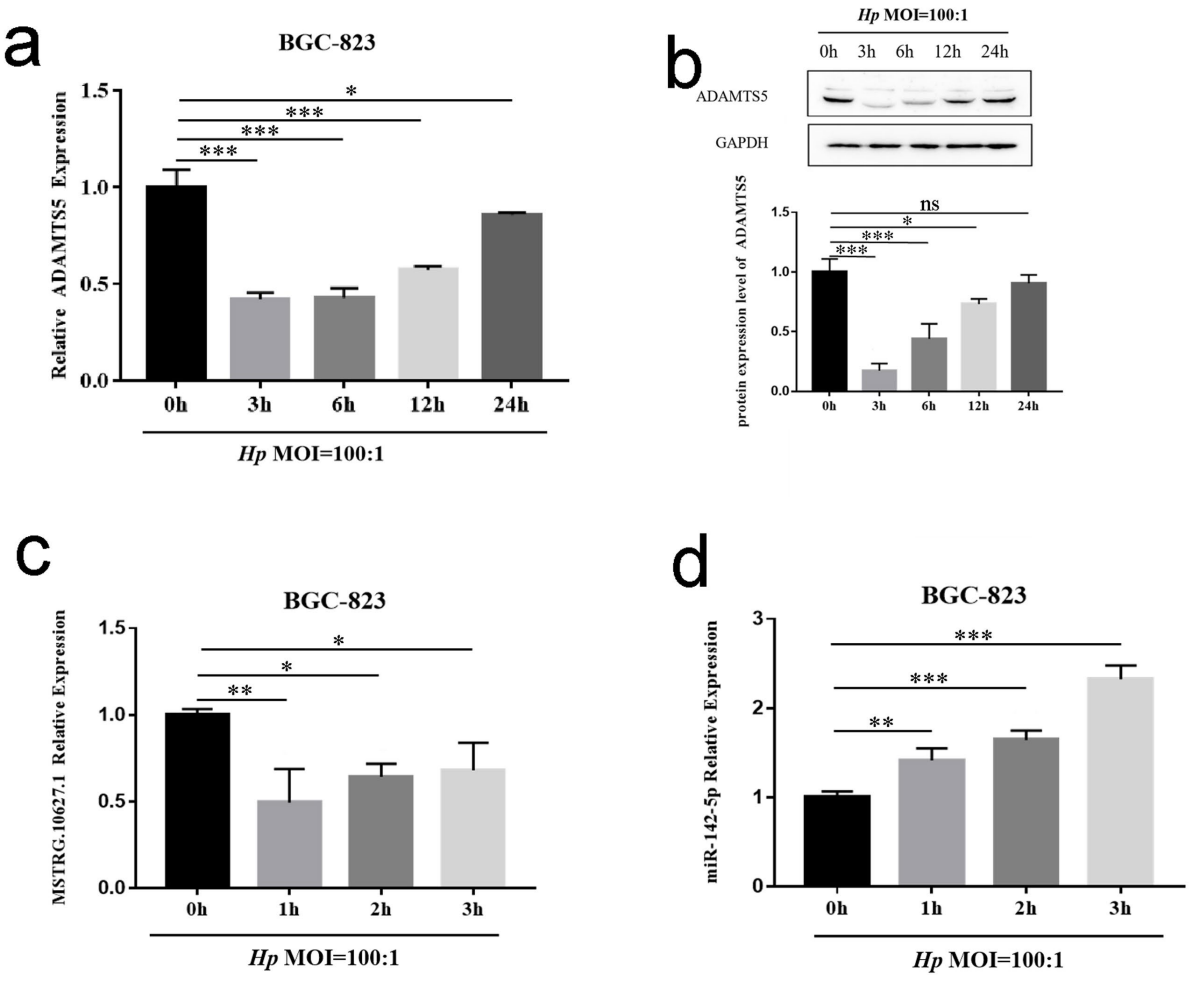
The change of lncRNA MSTRG.10627.1-miR-142-5p-mRNA (ADAMTS5) regulatory network in BGC-823 cell line after *H. pylori* infection. **(a–d)** The expression of ADAMTS5 mRNA **(a)**, ADAMTS5 protein **(b)**, lncRNA MSTRG.10627.1 **(c)**, and miR-142-5p **(d)** in the BGC-823 cell line after *H. pylori* infection. (Mean ± SD, *n* = 3. *p < 0.05, **p < 0.01, and ***p < 0.001).

ADAMTS5 mRNA expression decreased after the cells were infected with *H. pylori*, and the most significant decrease occurred after 3 h of infection (Figures 2a,b, *p* < 0.05). So, the time point was ultimately selected for subsequent experiments. Based on the above GC lncRNA-miRNA-mRNA regulatory network, the lncRNA (MSTRG.10627.1)-miRNA (miR-142-5p)-mRNA (ADAMTS5) regulatory network was selected and verified in subsequent experiments. The expressions of MSTRG.10627.1 and miR-142-5p of BGC-823 were reduced after the infection of *H. pylori* (Figures 2c,d, *p* < 0.05).

### MSTRG.10627.1 may negatively regulate miR-142-5p through the ceRNA and then promote the expression of ADAMTS5

**Figure 3:**
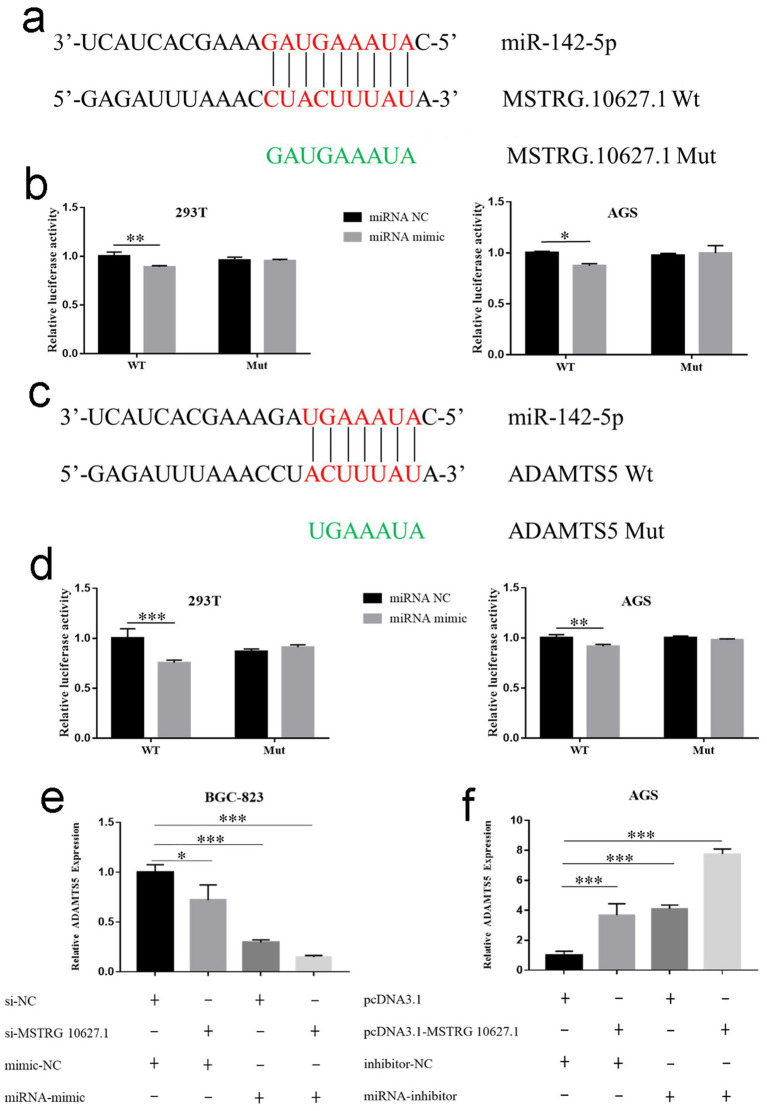
The detection of the targeted binding of MSTRG.10627.1 and miR-142-5p, as well as the target binding between miR-142-5p and mRNA (ADAMTS5), using a double luciferase reporter. **(a)** Schematic diagram of miR-142-5p binding sites and mutation sites in the 3′ UTR region of MSTRG.10627.1. **(b)** The targeted binding between MSTRG.10627.1 and miR-142-5p in 293T or AGS cell lines. **(c)** Schematic diagram of miR-142-5p binding sites and mutation sites in the 3′ UTR region of ADAMTS5. **(d)** The targeted binding between miR-142-5p and ADAMTS5 mRNA in 293T and AGS cell lines. **(e)** The expression of ADAMTS5 mRNA was detected by using qRT-PCR after co-transfection of si-MSTRG10627.1, si-NC, and miR-142-5p mimetics in the BGC-823 cell line. **(f)** After co-transfection with MSTRG.10627.1 plasmid, empty plasmid, miR-142-5p inhibitor, and inhibitor NC in AGS GC cell line, qRT-PCR was used to detect the expression of ADAMTS5 mRNA. (Mean ± SD, *n* = 3. *p < 0.05, **p < 0.01, and ***p < 0.001).

When the expression of miR-142-5p was up-regulated, the luciferase activity of the wild-type reporter gene was inhibited (Figures 3a,b; *p* < 0.05), while the luciferase activity of the mutant gene was not changed (Figures 3a,b, *p* > 0.05). The luciferase activity of the wild-type reporter gene was inhibited (Figures 3c,d, *p* < 0.01), and the luciferase activity of the mutant gene was not changed due to the overexpression of miR-142-5p (Figures 3c,d, *p* > 0.05), which indicated that the binding site on the 3′ UTR of ADAMTS5 directly bound to miR-142-5p. Compared with the transfected negative control, the ADAMTS5 expression in the other three groups was significantly reduced (Figure 3e, *p* < 0.05), whereas the expression of ADAMTS5 showed the most significant decrease in the group that interfered with MSTRG.10627.1 and was transfected with miR-142-5p mimic (Figure 3e, *p* < 0.05). Compared with the negative control group, the ADAMTS5 expression in the other three groups was significantly increased (Figure 3f; *p* < 0.05), while the ADAMTS5 expression was most significantly increased in the group that was transfected with the MSTRG.10627.1 eukaryotic expression vector and miR-142-5p inhibitor (Figure 3f, *p* < 0.001).

### The subcellular localization of lncRNA MSTRG.10627.1 in AGS cell line

**Figure 4:**
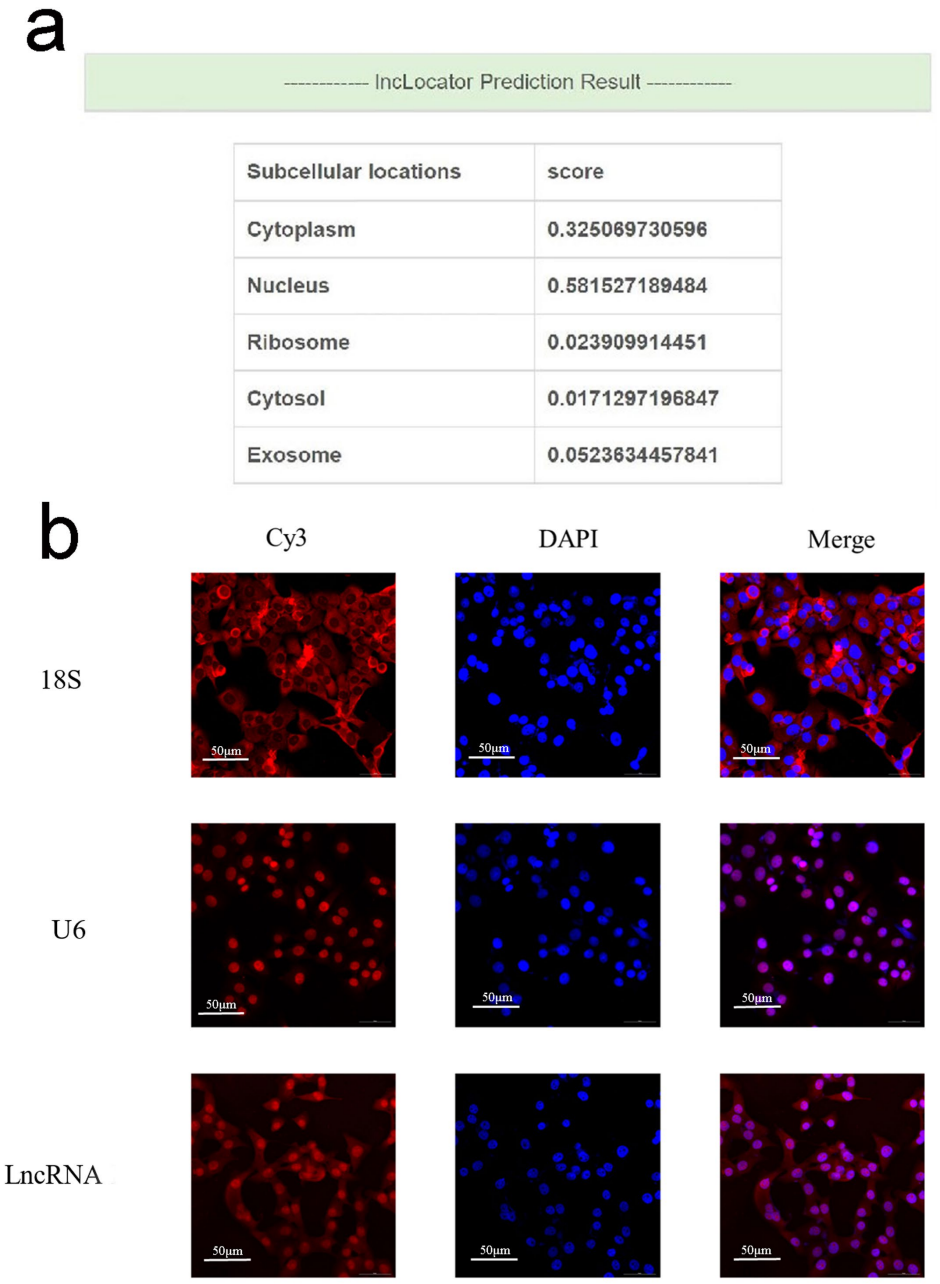
The localization of lncRNA MSTRG.10627.1 in the BGC-823 cell line. **(a)** The prediction of subcellular localization of lncRNA MSTRG.10627.1. **(b)** FISH was used to verify the subcellular localization of lncRNA MSTRG.10627.1. 18S and U6 are internal references. lncRNA and internal reference are labeled with red Cy3 tags; the nucleus is stained with blue DAPI. (Mean ± SD, *n* = 3).

The online prediction results showed that lncRNA MSTRG.10627.1 was distributed in the cytoplasm and nucleus (Figure 4a). The result of the FISH experiment indicated that lncRNA MSTRG.10627.1 was found in the cytoplasm and nucleus (Figure 4b), which was consistent with the prediction results.

### The effect of ADAMTS5 on GC cell lines

**Figure 5:**
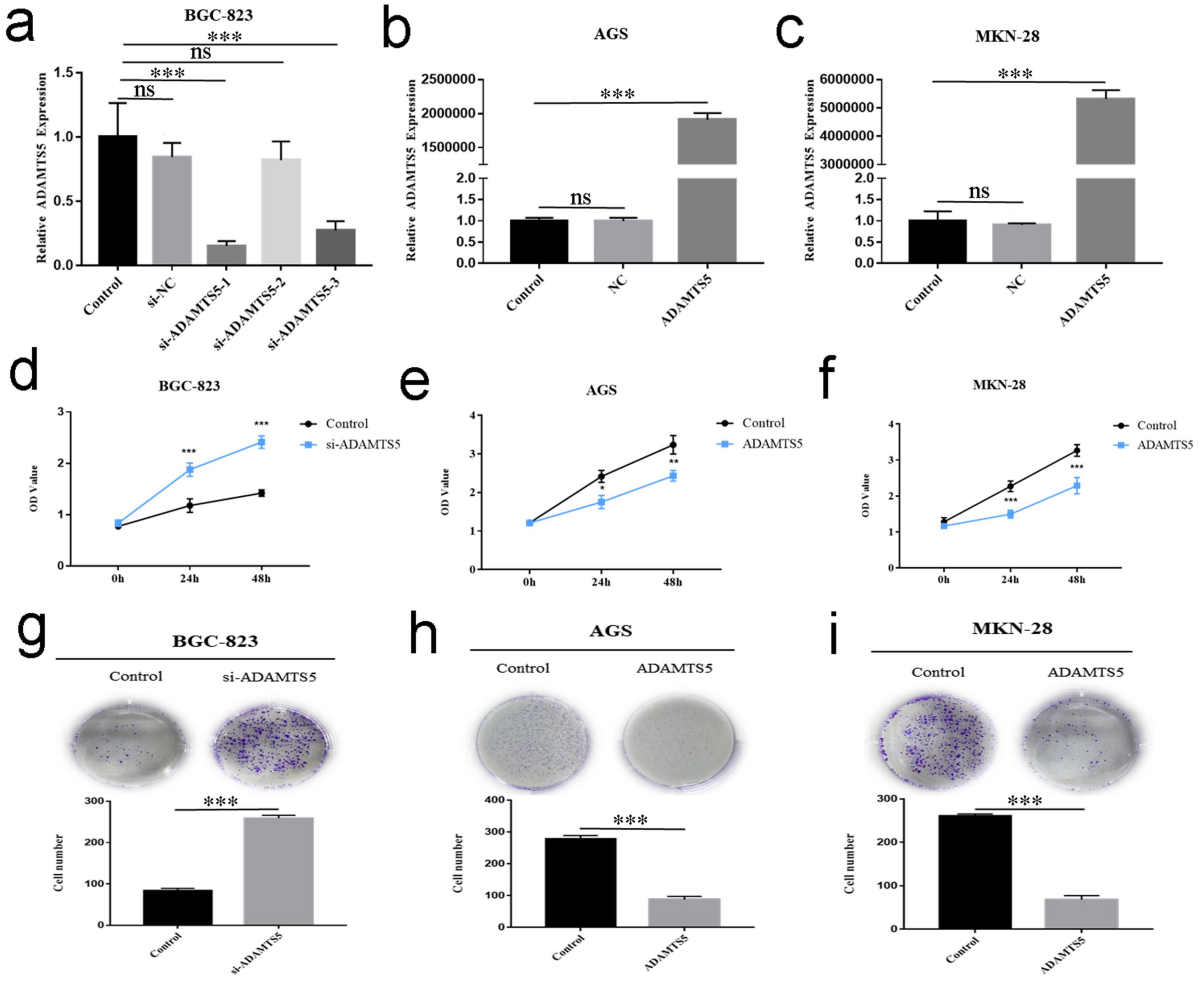
The effect of ADAMTS5 on the GC cell line proliferation and clonogenic ability. **(a)** The expression of ADAMTS5 mRNA in the BGC-823 cell line after transfection with siRNA. **(b,c)** The level of ADAMTS5 mRNA in AGS **(b)** and MKN-28 **(c)** cell lines after transfected with pADAMTS5. **(d)** The CCK-8 experiment detected the effect of ADAMTS5 on the proliferation ability of the BGC-823 cell line with ADAMTS5 silently expressed. **(e,f)** The proliferation ability of the AGS **(e)** and MKN-28 **(f)** cell lines with ADAMTS5 overexpressed. **(g)** The clone formation experiment was used to detect the effect of ADAMTS5 on the BGC-823 cell line clonogenic ability, with ADAMTS5 silently expressed. **(h,i)** The clonogenic ability of AGS **(h)** and MKN-28 **(i)** cell lines with ADAMTS5 overexpressed (Mean ± SD, *n* = 3. ^ns^*p* > 0.05, *p < 0.05, **p < 0.01, ***p < 0.001).

The quantitative reverse transcription PCR (qRT-PCR) results showed that the siRNA1, 3 of ADAMTS5 significantly reduced the ADAMTS5 expression in BGC-823 cells (Figure 5a) (*p* < 0.001), and the eukaryotic expression vector of ADAMTS5 significantly increased the expression of ADAMTS5 in AGS and MKN-28 (Figures 5b,c, *p* < 0.001). The CCK-8 showed that the cells transfected with the ADAMTS5 eukaryotic expression vector exhibited decreased proliferation capacity compared to the empty vector-transfected cells (Figures 5d,e, *p* < 0.05). However, the cells showed a significant increase in cell proliferation after silencing the expression of ADAMTS5 with small interfering RNA (Figure 5f, *p* < 0.05). When ADAMTS5 expression was interfered with, the number of cell clones increased significantly (Figure 5g, *p* < 0.001). Compared to the empty vector-transfected group, the number of cell clones was significantly reduced after transfection of the ADAMTS5 eukaryotic expression vector (Figures 5h,i, *p* < 0.001). The results showed that ADAMTS5 significantly inhibited the cloning ability of GC cell lines.

**Figure 6:**
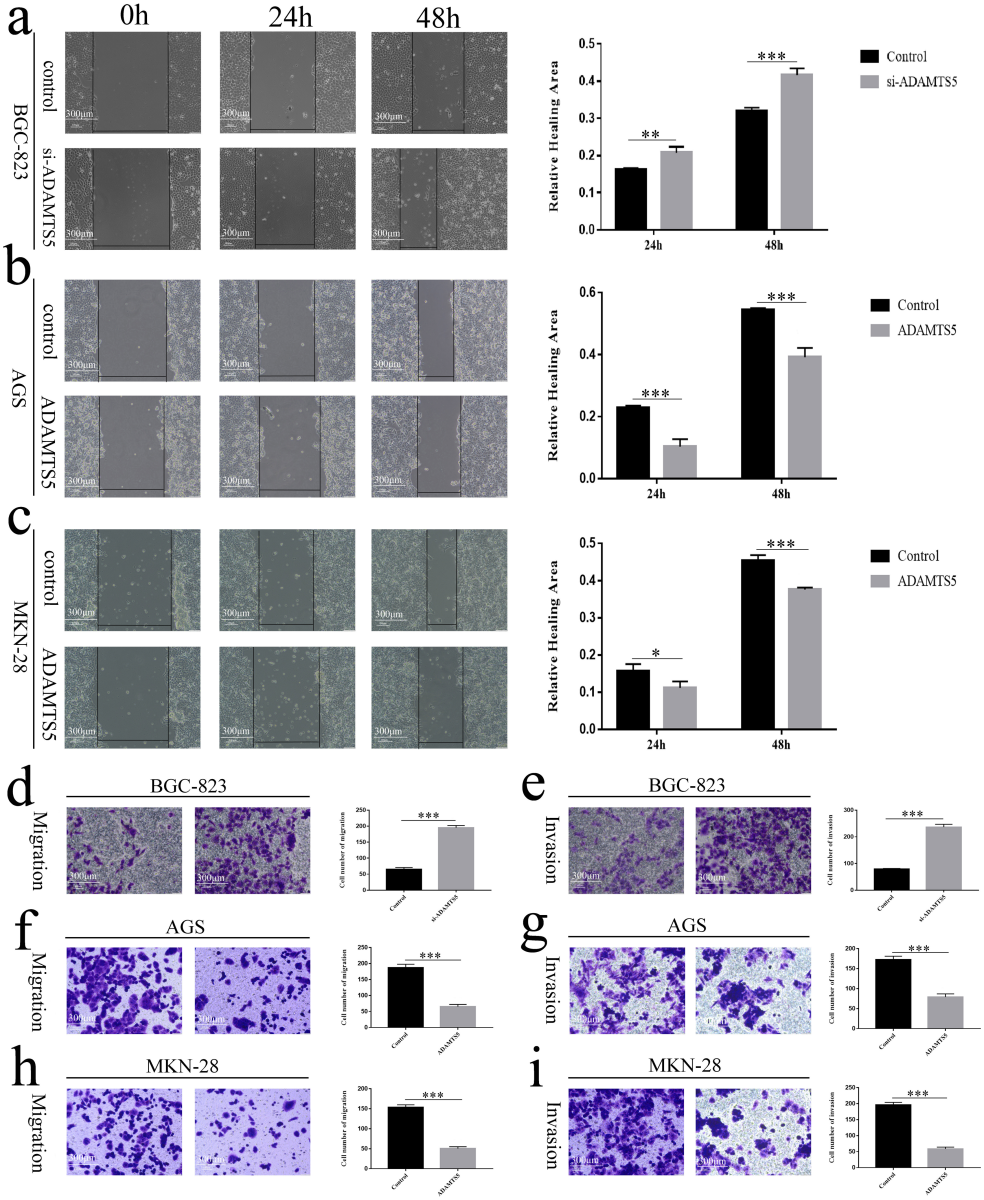
The effects of ADAMTS5 on the migration and invasion of GC cell lines. **(a)** The effect of ADAMTS5 on the migration ability of the BGC-823 cell line with ADAMTS5 silently expressed. **(b,c)** The effect of ADAMTS5 on the migration ability of the AGS **(b)** and MKN-28 **(c)** cell lines with ADAMTS5 overexpressed. **(d,e)** The effect of ADAMTS5 on the migration and invasion ability of the BGC-823 cell line with ADAMTS5 silenced. **(f,g)** The effect of ADAMTS5 on the migration and invasion ability of the AGS cell line with ADAMTS5 overexpressed. **(h,i)** The effect of ADAMTS5 on the migration and invasion ability of the MKN-28 cell line with ADAMTS5 overexpressed. (Mean ± SD, *n* = 3. ^ns^*p* > 0.05, *p < 0.05, **p < 0.01, and ***p < 0.001).

When ADAMTS5 was interfered with, the healing speed of cell scratches increased (Figure 6a, *p* < 0.01). The cell scratch experiment showed that cell scratch healing of the ADAMTS5-overexpressed group was significantly reduced compared to the empty vector group (Figures 6b,c, *p* < 0.05). The transwell experiment indicated that the number of cells passing through the upper compartment membrane and matrix glue increased significantly (Figures 6d,e, *p* < 0.001) when ADAMTS5 expression was interfered with. The number of cells that passed through the upper ventricular membrane and matrix glue in the ADAMTS5-overexpressed group decreased compared to the empty vector group (Figures 6f–i, *p* < 0.001). The results showed that ADAMTS5 significantly inhibited the migration and invasion ability of GC cells and may participate in regulating the metastasis of GC cells.

### ADAMTS5 affected PI3K/AKT/p53 pathway

**Figure 7:**
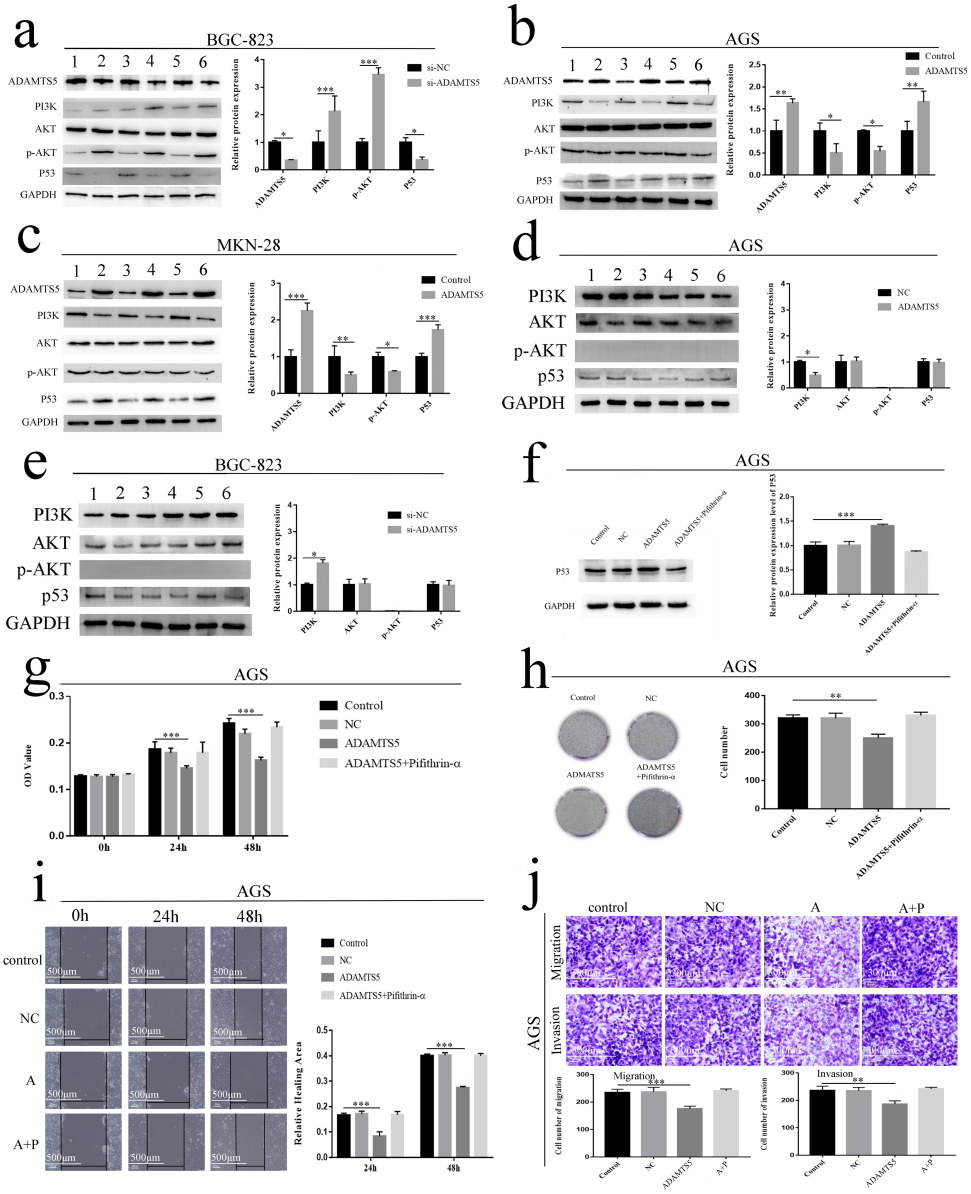
The effect of ADAMTS5 on the PI3K/AKT/p53 pathway. **(a)** The protein expression of ADAMTS5, PI3K, AKT, p-AKT, and p53 in the BGC-823 cell line with ADAMTS5 silently expressed. Lanes 1, 3, 5: control; lanes 2, 4, 6: si-ADAMTS5. **(b,c)** The protein expression of ADAMTS5, PI3K, AKT, p-AKT, and p53 in the AGS **(b)** and MKN-28 **(c)** cell line with ADAMTS5 over expressed. Lanes 1, 3, 5: control; lanes 2, 4, 6: ADAMTS5. **(d)** The protein expression of PI3K, AKT, p-AKT, and p53 in the AGS cells (treated with PI3K/AKT inhibitor) with ADAMTS5 over expressed. Lanes 1–3: control; lanes 4–6: ADAMTS5 overexpressed. **(e)** The protein expression of PI3K, AKT, p-AKT, and p53 in the BGC-823 cells (treated with PI3K/AKT inhibitor) with ADAMTS5 down-expressed. Lanes 1–3: control; lanes 4–6: si-ADAMTS5. **(f)** The protein expression of ADAMTS5 in the AGS cell line with pifithrin-α treatment. **(g,h)** The proliferation **(g)** and clonality **(h)** ability of AGS cell line with pifithrin-α treatment. **(i)** The migration ability of AGS cell line with pifithrin-α treatment. A: ADAMTS5; A + P: ADAMTS5 + pifithrin-α. **(j)** The migration and invasion ability of AGS cell line with pifithrin-α treatment. A: ADAMTS5; A + P: ADAMTS5 + pifithrin-α. (Mean ± SD, *n* = 3. *p < 0.05, **p < 0.01, and ***p < 0.001).

After the ADAMTS5 silencing expression with siRNA, western blot results showed the expression of PI3K protein (Figure 7a, *p* < 0.001), and the phosphorylation of downstream AKT protein was increased (Figure 7a, *p* < 0.001), while p53 protein expression was decreased in the cells (Figure 7a, *p* < 0.05). However, the overexpressed ADAMTS5 reduced PI3K protein expression and AKT protein phosphorylation (*p* < 0.05), and increased p53 expression, a tumor suppressor factor (Figures 7b,c, *p* < 0.01). In the overexpressed ADAMTS5 cells, PI3K protein expression was reduced after treatment with the PI3K/AKT inhibitor (*p* < 0.05), but no AKT protein phosphorylation was detected because of the inhibitor, and the levels of p53 did not change (*p* > 0.05) when compared with control cells (Figure 7d). The down-expressed ADAMTS5 improved PI3K protein expression (*p* < 0.05), but no AKT protein phosphorylation was detected because of the inhibitor; the levels of p53 had no change (*p* > 0.05) when compared with control cells (Figure 7e). The results suggested that ADAMTS5 may activate the expression of tumor suppressor factor p53 by inhibiting the PI3K/AKT signaling pathway.

Western blot results showed that p53 protein expression was significantly inhibited after the treatment of pifithrin-α (*p* < 0.001) (Figure 7f). After treatment with pifithrin-α, the roles of ADAMTS5 in inhibiting the GC cells’ proliferation (Figure 7g, *p* < 0.001), clone formation (Figure 7h, *p* < 0.01), migration, and invasion were eliminated (Figures 7i,j, *p* < 0.01). The above results suggested that ADAMTS5 may play an inhibitory role in GC cells by activating p53.

### The antitumor effect of ADAMTS5 *in vivo*

**Figure 8:**
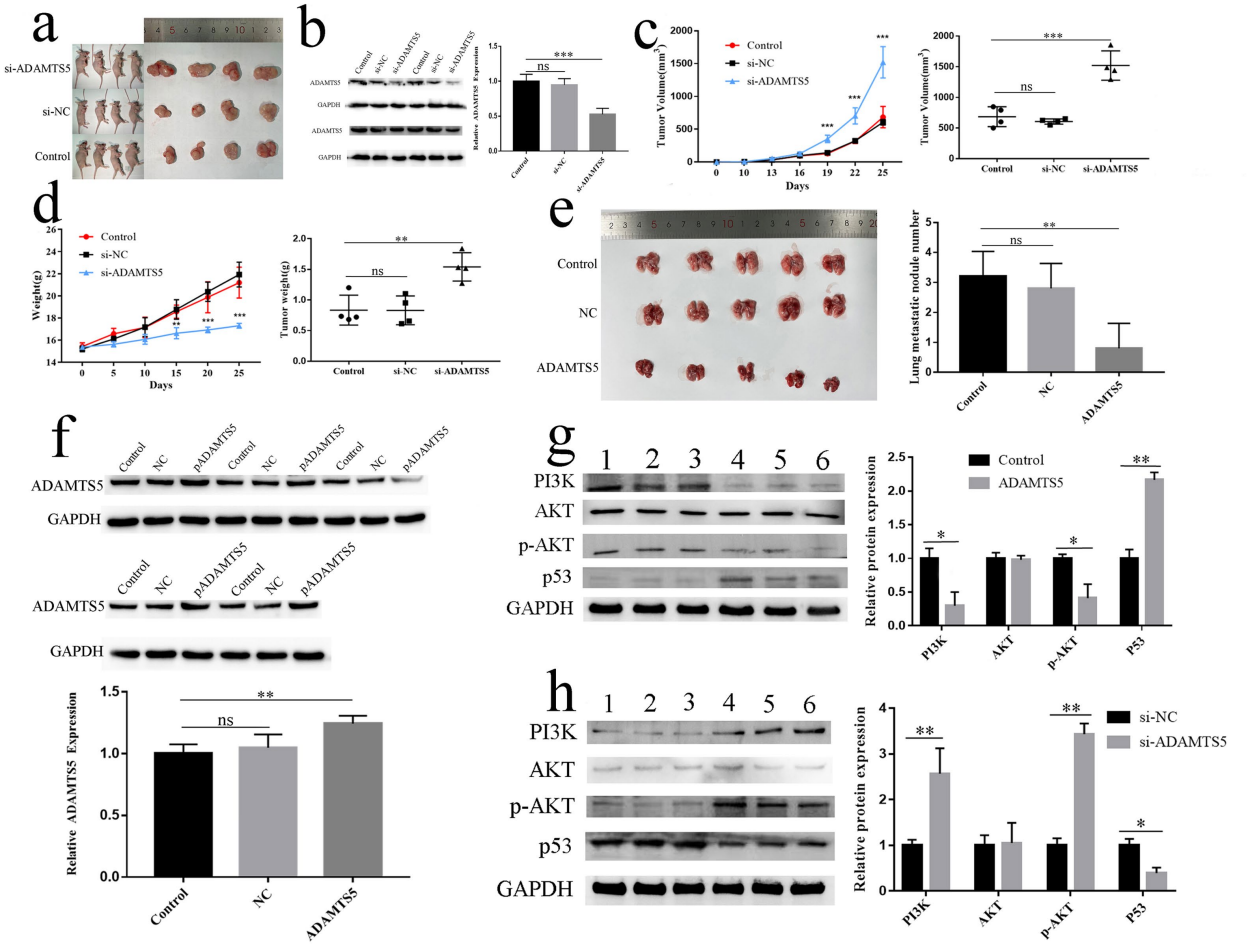
The effect of ADAMTS5 in nude mice. **(a)** Subcutaneous tumor model in nude mice and the tumors from control, si-NC, and si-ADAMTS5 groups. **(b)** The protein expression of ADAMTS5 from tumors in control, si-NC, and si-ADAMTS5 mice. **(c,d)** The volume **(c)** and weight **(d)** of tumors in control, si-NC, and si-ADAMTS5 mice. **(e)** Metastatic tumor model in nude mice and the number of lung metastases. **(f)** The protein level of ADAMTS5 from tumors in control, NC, and ADAMTS5 mice. **(g)** The protein expression of PI3K, AKT, p-AKT, and p53 in the tumors with ADAMTS5 over expressed. Lanes 1–3: control; lanes 4–6: ADAMTS5 overexpressed. **(h)** The protein expression of PI3K, AKT, p-AKT, and p53 in the tumors with ADAMTS5 down-expressed. Lanes 1–3: control; lanes 4–6: si-ADAMTS5. (Mean ± SD, *n* = 3. ^ns^*p* > 0.05, *p < 0.05, **p < 0.01, and ***p < 0.001).

The subcutaneous tumor formation model was successfully constructed (Figure 8a). The ADAMTS5 protein was significantly reduced in the small interfering RNA-transfected mice (*p* < 0.001), demonstrating successful *in vivo* transfection (Figure 8b). Compared to the control group, the tumor volume (Figure 8c, *p* < 0.001) and weight (Figure 8d, *p* < 0.05) in the si-ADAMTS5 group were significantly increased. The body weight of the nude mice in the si-ADAMTS5 group decreased compared with the control group (Figure 8d, *p* < 0.05), while the tumor volume change curve in nude mice was opposite (Figure 8c, *p* < 0.001). The results indicated that the growth rate of tumors significantly increased after silencing ADAMTS5 expression *in vivo*.

The lung tissue and metastatic lesions in the ADAMTS5-overexpressed group were smaller and fewer than in the blank control and negative control groups (Figure 8e, *p* < 0.05). Western blot showed that the expression of ADAMTS5 in the lung tissue of the overexpression group was up-regulated compared with the control group (Figure 8f, *p* < 0.05), indicating that the ADAMTS5-overexpressed GC cells still stably expressed ADAMTS5 *in vivo*. In tumors, overexpression of ADAMTS5 reduced PI3K protein expression (*p* < 0.05). AKT protein phosphorylation was reduced, while p53 levels were improved compared with control tumors (*p* < 0.05) (Figure 8g). The down-expressed ADAMTS5 improved PI3K protein and AKT protein phosphorylation expression (*p* < 0.05), while p53 had been reduced when compared with control tumors (*p* < 0.05) (Figure 8h).

## Discussion

The cure rate of early GC is relatively high, while it is low in the middle and late stages. The diagnosis of early GC is challenging, underscoring the urgent need for new diagnostic biomarkers and therapeutic targets. In this study, full transcriptome sequencing of six GC cell lines was performed, and the differentially expressed genes were identified to construct an lncRNA-miRNA-mRNA ceRNA regulatory network of GC. The lncRNA (MSTRG.10627.1)—miRNA (miR-142-5p)—mRNA (ADAMTS5) regulatory axis was selected out in *H. pylori*-infected GC cells.

lncRNA, which was especially localized in the cytoplasm, reduced the expression of miRNA by regulating miRNA response elements and then reduced the inhibitory effect of corresponding miRNAs on the target gene mRNAs (Zhu et al., 2022). lncRNA TUG1 regulated miR-1-3p to promote cell proliferation during hepatocarcinogenesis (Tang et al., 2022). lncRNA RPSAP52 promoted cell proliferation and inhibited apoptosis by regulating miR-665/STAT3 in GC (He et al., 2022). Our results indicated that MSTRG.10627.1 was mainly localized in the cytoplasm and nucleus of GC cells, suggesting it might play some role in regulating the corresponding miRNAs. The targeted binding relationships between MSTRG.10627.1 and miR-142-5p and between miR-142-5p and ADAMTS5 were verified by dual luciferase reporter gene experiments. Gene-knockout and gene-overexpression experiments indicated that MSTRG.10627.1 negatively regulated miR-142-5p, which in turn regulated the expression of the target gene ADAMTS5. Our study first found that *H. pylori* might regulate ADAMTS5 expression in GC cell lines by lncRNA MSTRG.10627.1, which negatively regulates miR-142-5p. Although the direct targeting relationship between MSTRG.10627.1 and miR-142-5p, as well as between miR-142-5p and ADAMTS5, had been verified in this study, direct regulation of non-coding RNAs (MSTRG.10627.1/miR-142-5p) to recapitulate the ADAMTS5-mediated phenotypic effects may be verified in further experiments. For example, in the background of normal ADAMTS5 expression, overexpressed MSTRG.10627.1 or inhibited miR-142-5p was used to verify whether it could reverse the downregulation of ADAMTS5 induced by *H. pylori*. In summary, the present study verified the possibility of regulation of non-coding RNAs (MSTRG.10627.1/miR-142-5p) to recapitulate the ADAMTS5-mediated phenotypic effects.

ADAMTS5 plays different roles in different tumors. ADAMTS5 overexpression in liver cancer, colorectal cancer, and glioblastic multiform carcinoma played a role in promoting cancer (Akcora-Yildiz et al., 2022), while it inhibited the growth of mouse melanoma (Renganathan et al., 2018). Recent studies have shown that ADAMTS5 was down-regulated in GC tissue and inhibited tumor metastasis and angiogenesis by inhibiting ETS1-mediated changes in MVD (Huang et al., 2019). The low expression of ADAMTS5 was related to gender, histological type, degree of differentiation, M stage, TNM stage, and vascular invasion (Noorolyai et al., 2019). In our research, the AGS or MKN-28 cell of ADAMTS5 overexpression and the BGC-823 cell of silenced expression were constructed *in vitro*. The results showed that ADAMTS5 could inhibit the proliferation, migration, and invasion of GC cell lines, which suggested an inhibitory role in GC. The results were consistent with a previous study (Huang et al., 2019). It might serve as a new candidate prognostic marker and potential therapeutic target in GC.

The analysis of KEGG signaling pathway enrichment of the lncRNA-miRNA-mRNA regulatory network showed a significant enrichment in the PI3K/AKT signaling pathway. The PI3K/AKT signaling pathway is among the most commonly dysregulated pathways in cancer. It is essential for many cellular biological processes and plays a crucial role in promoting tumor genesis, progression, and therapeutic responses (Noorolyai et al., 2019; Hoxhaj and Manning, 2020). AKT is the main downstream target of PI3K and a serine threonine kinase derived from the oncogene family involved in tumorigenesis (Zhang et al., 2022). Activation of PI3K leads to AKT phosphorylation, and AKT is further activated at the cell membrane. Then, activated AKT can regulate the proliferation, angiogenesis, and promote migration of cancer cells (King et al., 2015). Activated AKT can phosphorylate p53 by its ubiquitin ligase MDM2, which inhibits cell cycle arrest and apoptosis caused by p53 (Zhang et al., 2022; Zou et al., 2019). To further explore the mechanism of ADAMTS5, the expression of PI3K/AKT signaling pathway proteins was detected in ADAMTS5-overexpressed and silenced GC cell lines. ADAMTS5 overexpression had the potential to inhibit the PI3K-AKT signaling pathway and to promote p53 expression, a downstream tumor suppressor molecule. Pifithrin-α, a specific inhibitor of p53 transcriptional activity, significantly boosted the activity and proliferation of SNU-216 and HGC-27 cells, reversing the inhibitory effect of si-UPK3A on these parameters (Xu et al., 2022). In our study, p53 inhibitor (pifithrin-α) significantly reduced the expression of p53 protein and reversed the antitumor effect of ADAMTS5. Pifithrin-α inhibits the transcriptional activation function of the p53 protein, but also suppresses the expression of heat shock protein HSP70. Therefore, pifithrin-α may have off-target effects, including potential confounding effects of inhibiting HSP70.

ADAMTS5 were up-regulated in proliferating glioblastoma cells and in head and neck cancer; the proteases might contribute to their invasive potential (Held-Feindt et al., 2006; Nakada et al., 2005; Viapiano et al., 2008; Demircan et al., 2009). Moreover, high expression of ADAMTS5 was considered to be a potent marker for lymphatic invasion and lymph node metastasis in colorectal cancer (Haraguchi et al., 2017), which seemed to contradict Li’s et al. (2018) study. However, in the study, ADAMTS5 was verified as a tumor suppressor in GC through nude mice *in vivo*. ADAMTS5 reduced the volume and weight of subcutaneous tumors and the number of pulmonary metastases. Furthermore, ADAMTS5 regulated the migration and invasion of non-small cell lung cancer (NSCLC), and it may be a useful therapy target in NSCLC (Gu et al., 2016). Epigenetic silencing of ADAMTS5 increased invasiveness and poor survival in colorectal cancer patients (Li et al., 2018). These studies suggested that ADAMTS5 inhibited these tumors, and our research confirmed this.

The dual role of ADAMTS5 in cancer (pro-cancer vs. anti-cancer) is not contradictory, but rather a concrete manifestation of its multifunctionality in the highly heterogeneous tumor biological context. The core lies in its ability to cleave specific substrates, producing fragments with different or even opposite biological activities.

In summary, *H. pylori* induces a decrease in ADAMTS5 expression in GC cells. The reduced ADAMTS5 expression promotes proliferation, invasion, and migration of GC cell lines. *H. pylori* may downregulate ADAMTS5 expression of GC cell lines through the MSTRG 10627.1 (lncRNA)—miR-142-5p (miRNA) axis. ADAMTS5 could act on the PI3K/AKT signaling pathway, thereby affecting the downstream tumor suppressor p53. The down-regulated ADAMTS5 expression promotes the development and metastasis of GC *in vivo*. Therefore, a novel regulatory axis was identified in the present study, which further elucidates the mechanism of GC induced by *H. pylori*. The findings may lay the foundation for discovering new candidate diagnostic markers and therapeutic targets for GC.

## Data Availability

The original contributions presented in the study are included in the article/Supplementary material, further inquiries can be directed to the corresponding authors.

## Footnotes

1
http://www.csbio.sjtu.edu.cn/
