# Supplementary figures and images for: Helicobacter pylori contributes to GC progression, possibly via the MSTRG.10627.1/miR-142-5p/ADAMTS5 pathway
Source: Front Microbiol. 2026 Feb 2;16:1686246. doi: 10.3389/fmicb.2025.1686246 (PMC12908916; doi:10.3389/fmicb.2025.1686246)

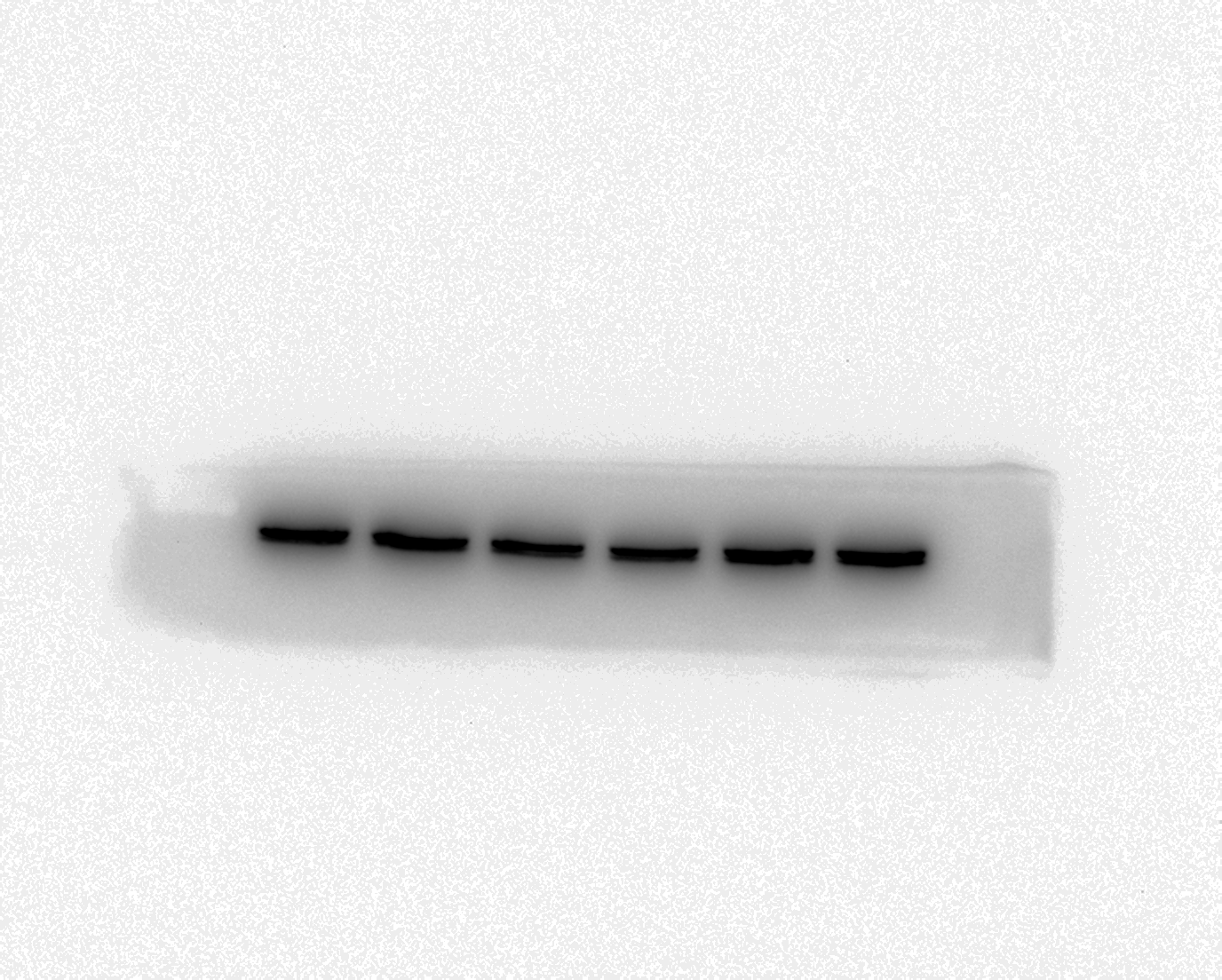

Supplement: Supplementary file 1 [file Presentation_1.zip › Supplementary materials/Image 1.tif]

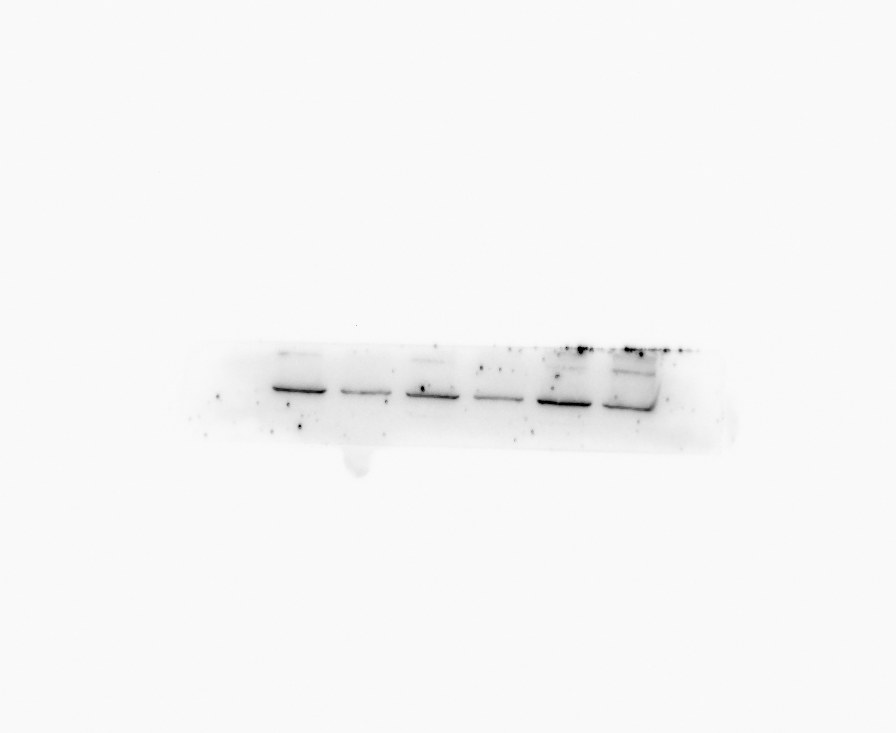

Supplement: Supplementary file 1 [file Presentation_1.zip › Supplementary materials/Image 10.tif]

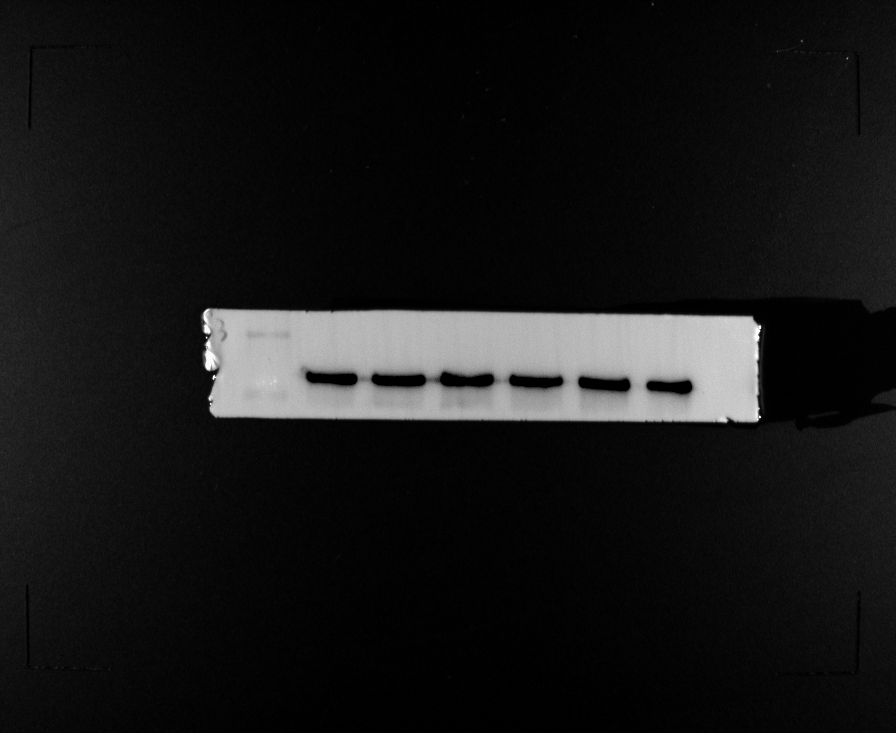

Supplement: Supplementary file 1 [file Presentation_1.zip › Supplementary materials/Image 11.tif]

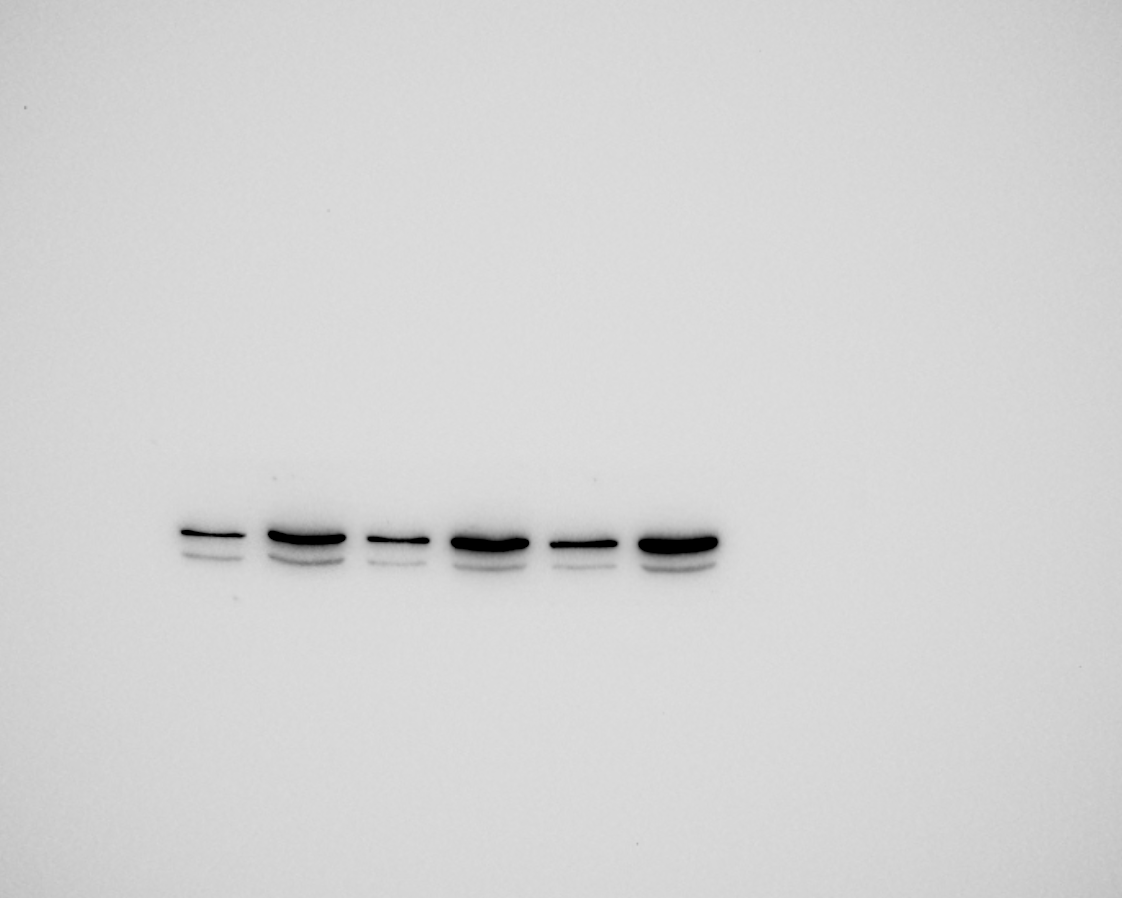

Supplement: Supplementary file 1 [file Presentation_1.zip › Supplementary materials/Image 12.tif]

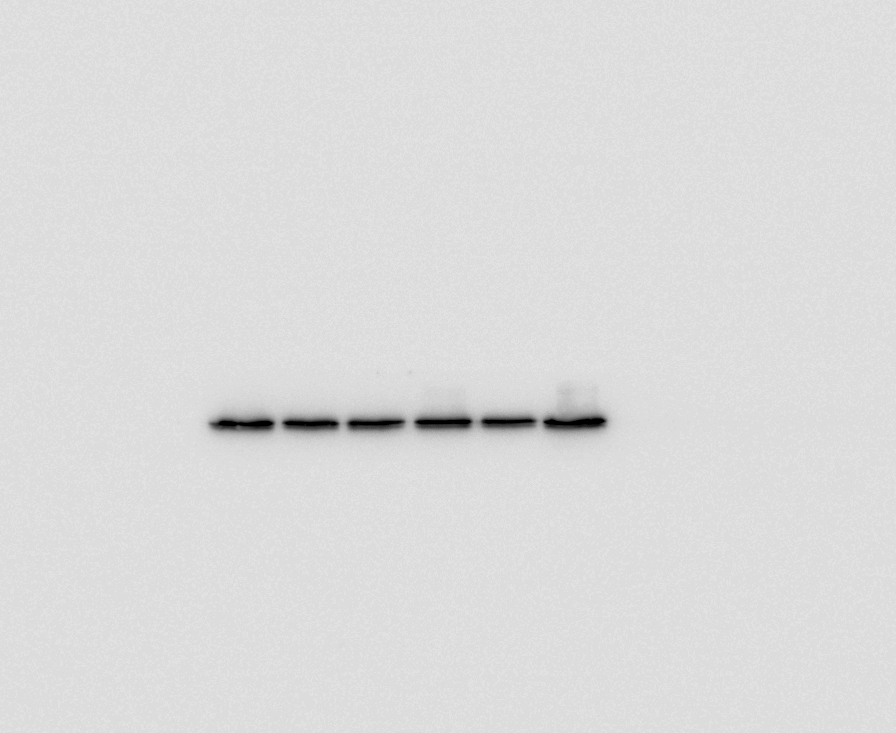

Supplement: Supplementary file 1 [file Presentation_1.zip › Supplementary materials/Image 13.tif]

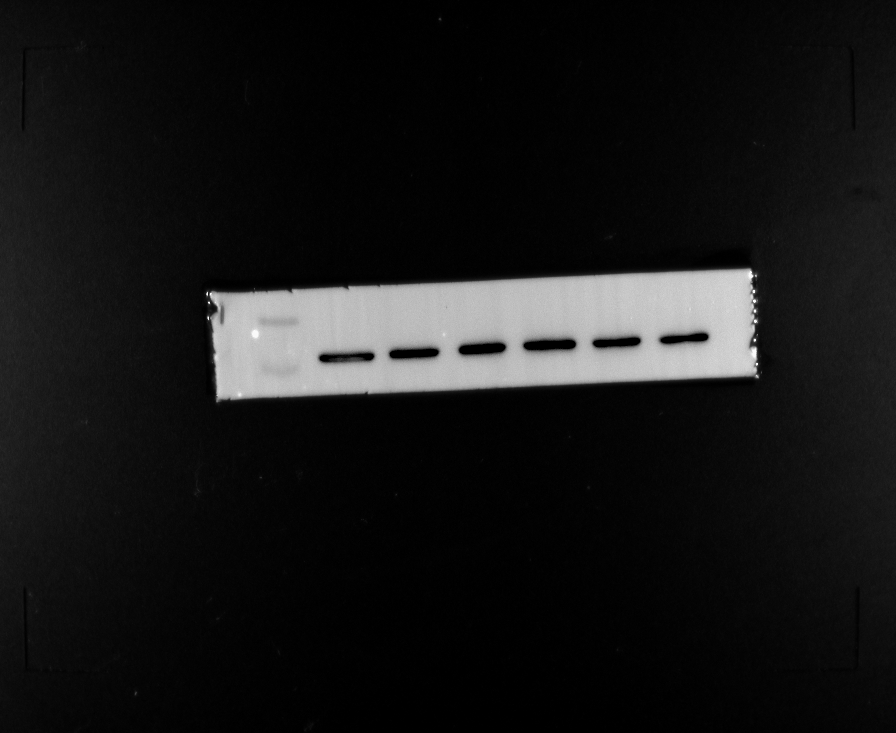

Supplement: Supplementary file 1 [file Presentation_1.zip › Supplementary materials/Image 14.tif]

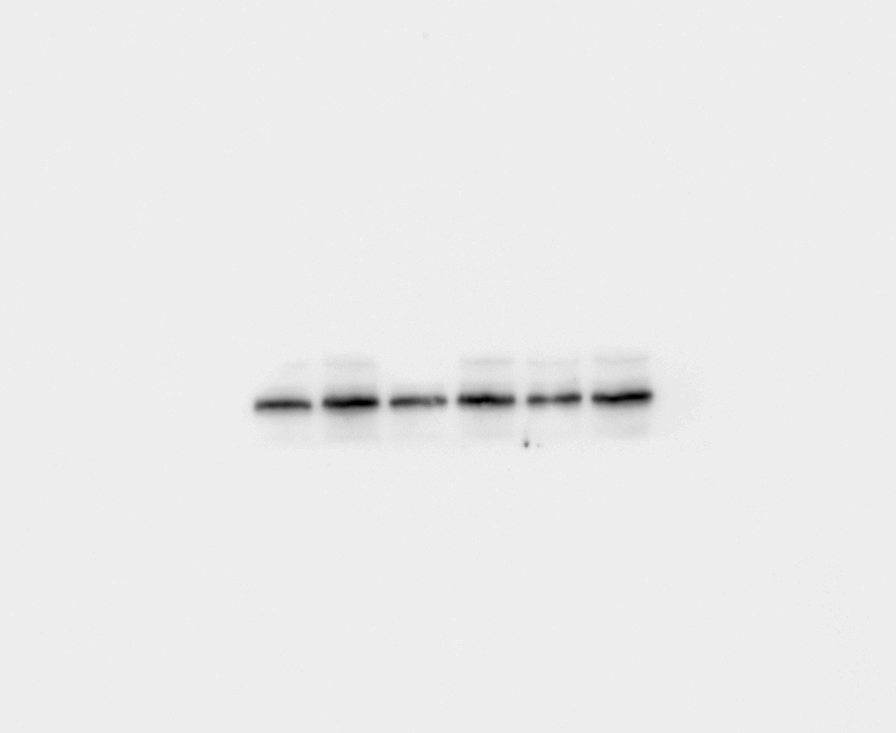

Supplement: Supplementary file 1 [file Presentation_1.zip › Supplementary materials/Image 15.tif]

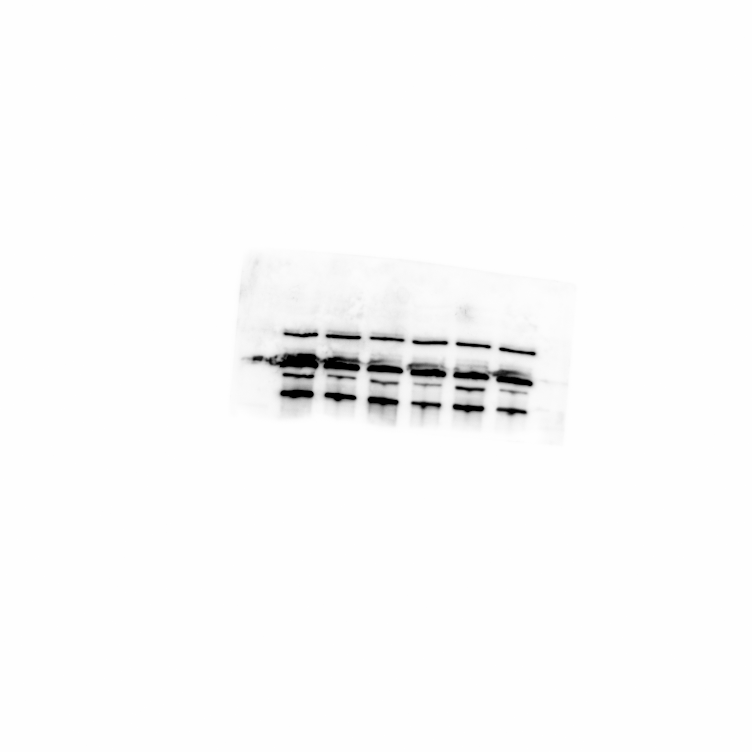

Supplement: Supplementary file 1 [file Presentation_1.zip › Supplementary materials/Image 2.tif]

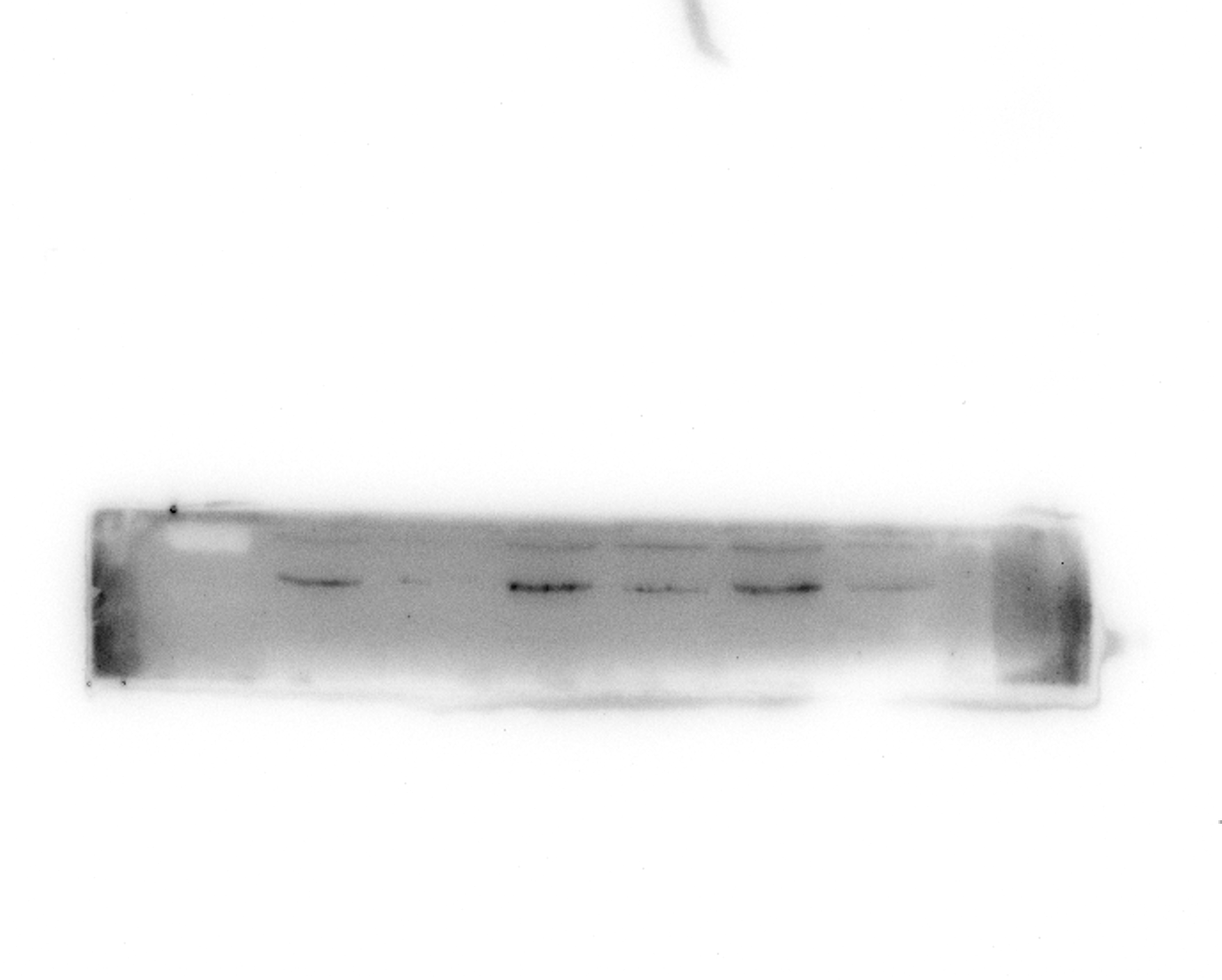

Supplement: Supplementary file 1 [file Presentation_1.zip › Supplementary materials/Image 3.tif]

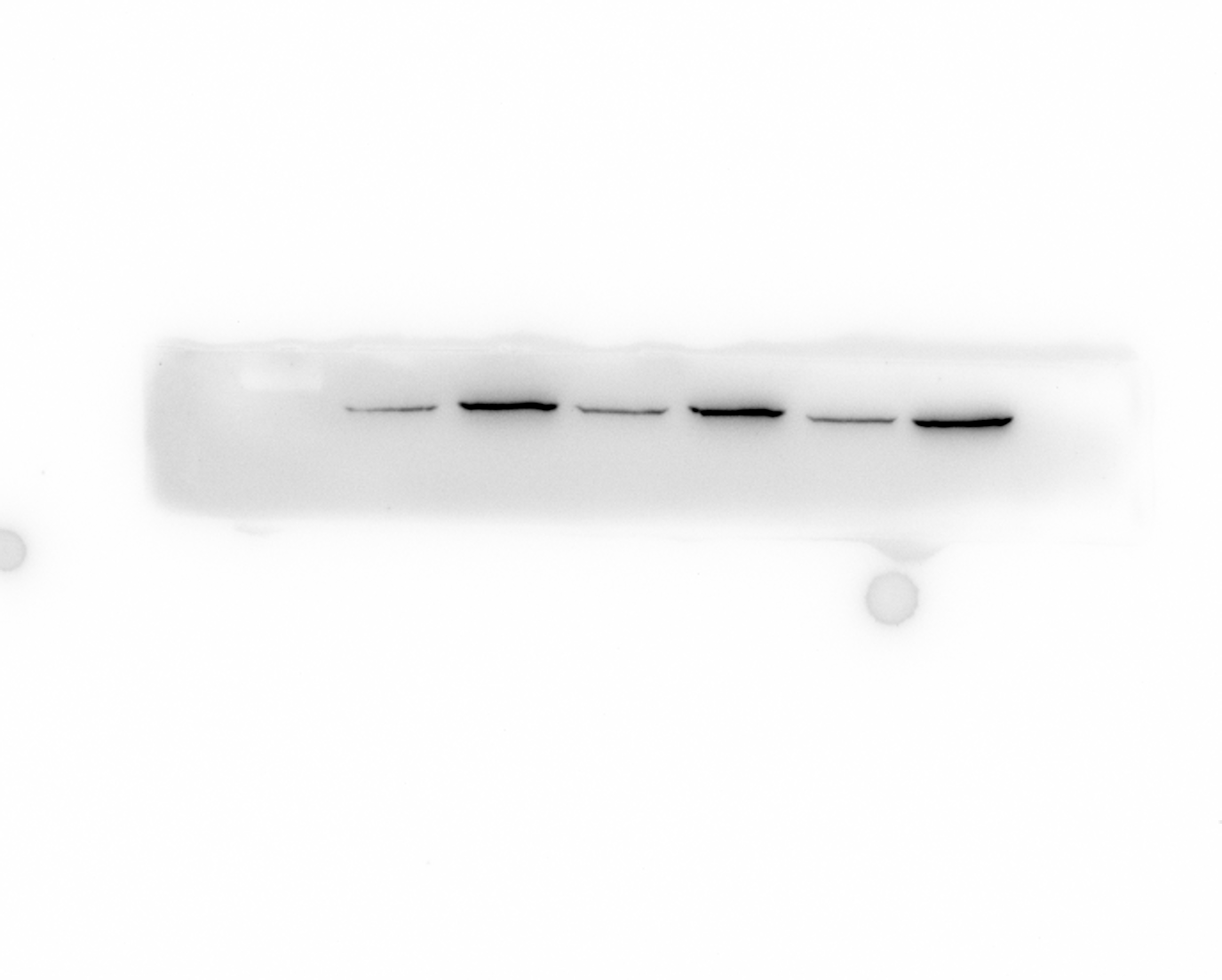

Supplement: Supplementary file 1 [file Presentation_1.zip › Supplementary materials/Image 4.tif]

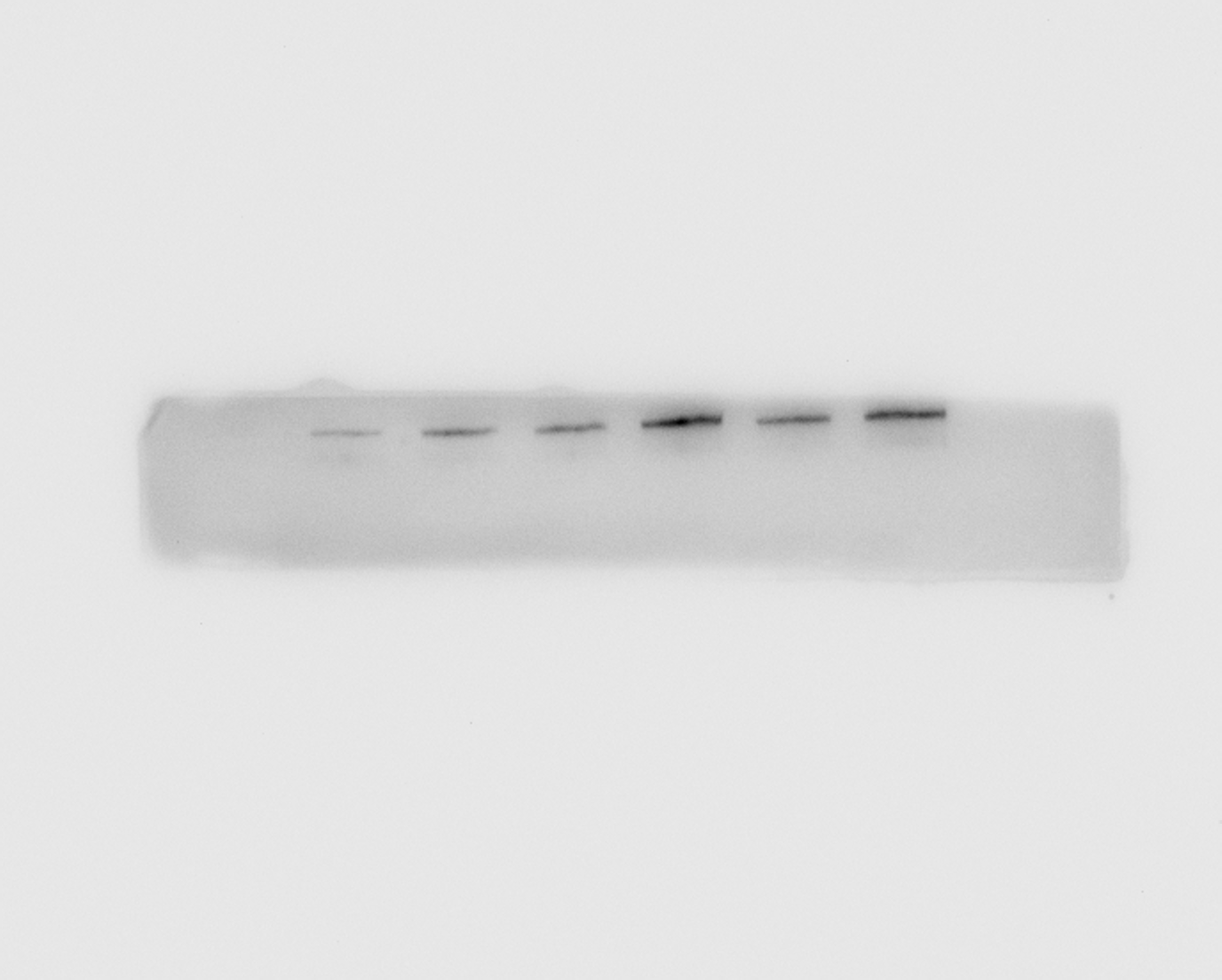

Supplement: Supplementary file 1 [file Presentation_1.zip › Supplementary materials/Image 5.tif]

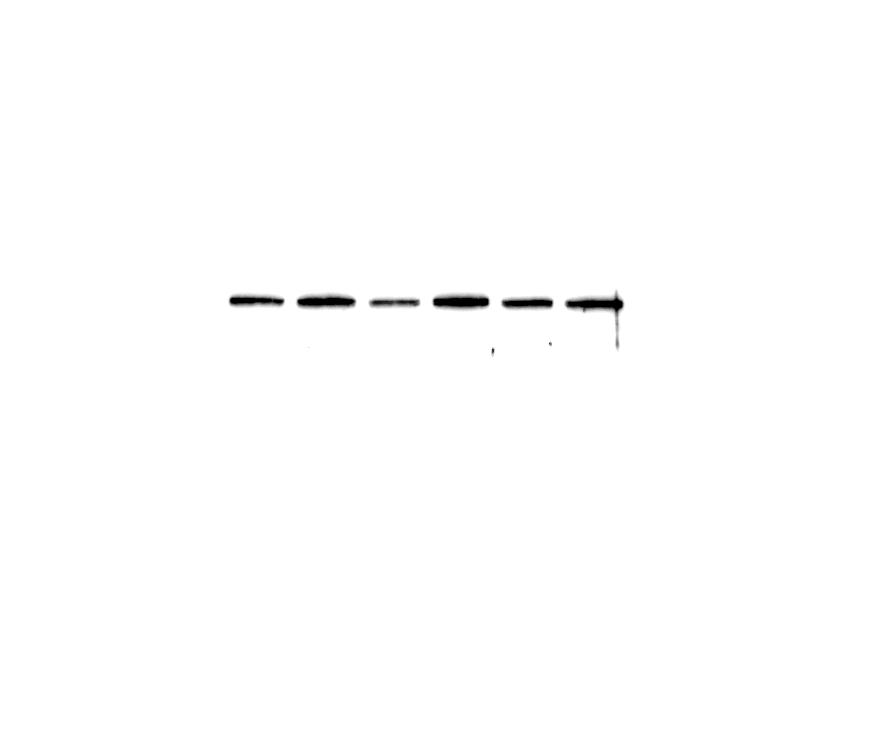

Supplement: Supplementary file 1 [file Presentation_1.zip › Supplementary materials/Image 6.tif]

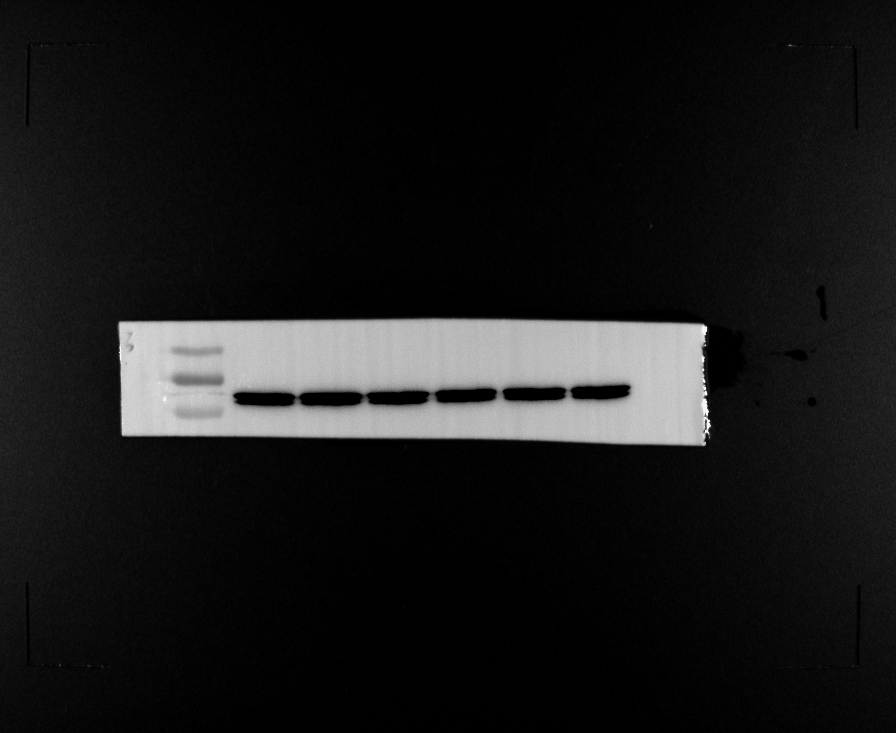

Supplement: Supplementary file 1 [file Presentation_1.zip › Supplementary materials/Image 7.tif]

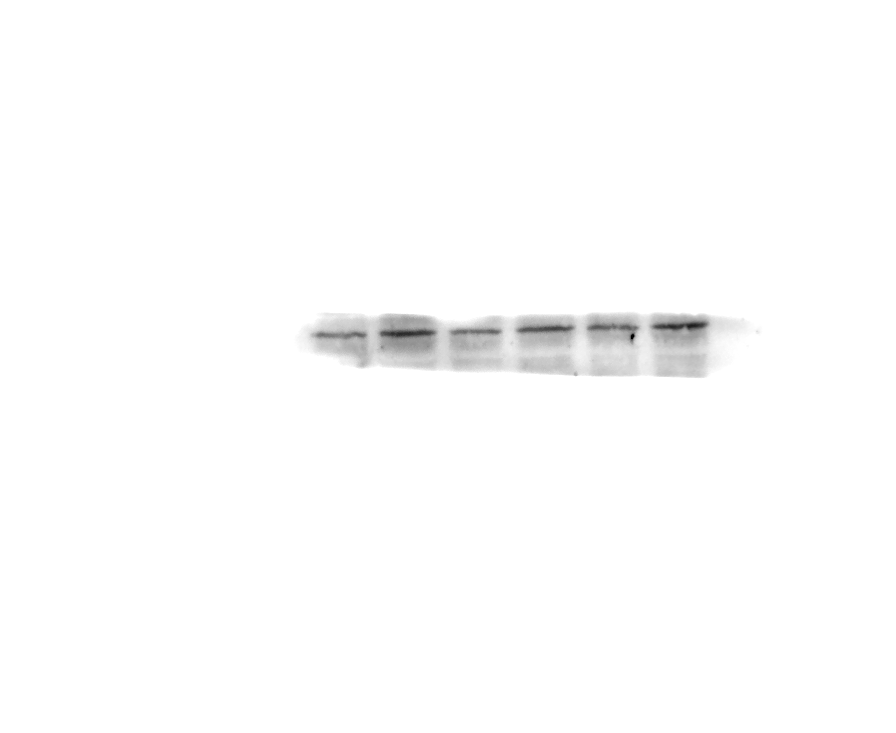

Supplement: Supplementary file 1 [file Presentation_1.zip › Supplementary materials/Image 8.tif]

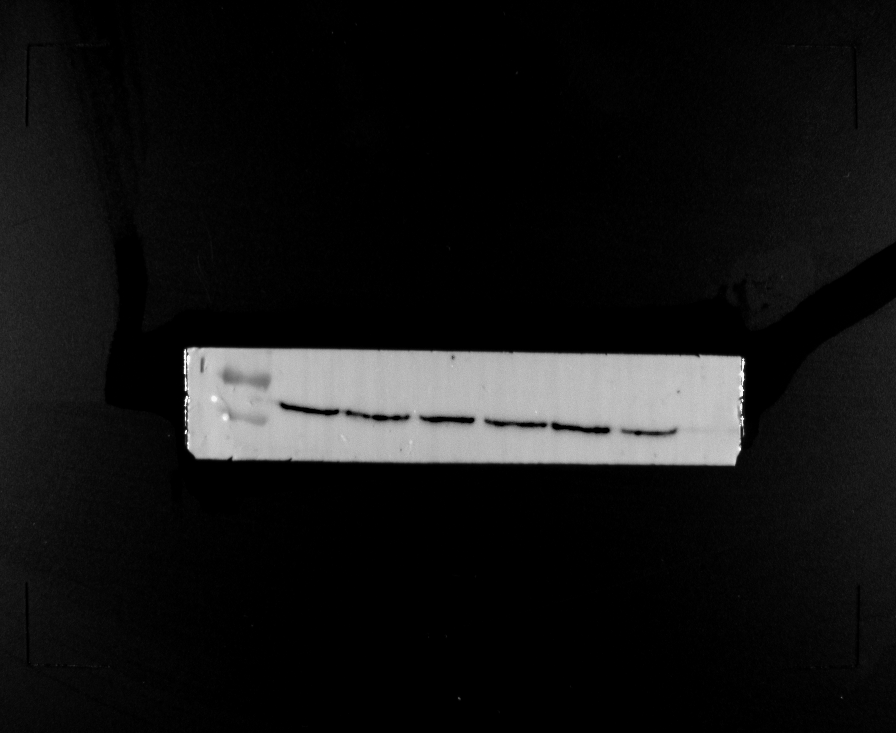

Supplement: Supplementary file 1 [file Presentation_1.zip › Supplementary materials/Image 9.tif]
