# Supplementary material for: Helicobacter pylori contributes to GC progression, possibly via the MSTRG.10627.1/miR-142-5p/ADAMTS5 pathway
Source: Front Microbiol. 2026 Feb 2;16:1686246. doi: 10.3389/fmicb.2025.1686246 (PMC12908916; doi:10.3389/fmicb.2025.1686246)
Supplement: Supplementary file 2 [file Supplementary_file_1.pdf]

Additional file 1: Table S1. The sequences of primer

Item	Sequence（5’ to 3’）
si-ADAMTS5-1	AAGCTCAAAGCTGCAGTATGA
	TCATACTGCAGCTTTGAGCCA
si-ADAMTS5-2	AAGGAGACAACTCCAGCTGTA
	TACAGCTGGAGTTGTCTCCCA
si-ADAMTS5-3	AAGTTGGAACCTTTAATAAGA
	TCTTATTAAAGGTTCCAACCA
si-MSTRG.10627.1-1	TTGCTCATAGTAGCACAGTCA
	TGACTGTGCTACTATGAGCCG
si-MSTRG.10627.1-2	TTGGATCAGTTAGACCTTCAT
	ATGAAGGTCTAACTGATCCCG
si-MSTRG.10627.1-3	TTGCTTCCAGTGTTCACATAA
	TTATGTGAACACTGGAAGCCG
miR-142-5p mimics	GCAUAAAGUAGAAAGCACUACU
	GAGUAGUGCUUUCUACUUUAUG
miR-142-5p inhibitor	AGUAGUGCUUUCUACUUUAUGC

Additional file 2: Table S2. LncRNA-miRNA-mRNA regulatory network genes in gastric cancer

LncRNA(17)	mRNA(16)	Common miRNA(32)
MSTRG.36280.1	SLC4A8	has-miR-577
MSTRG.37033.1	KYNU	has-miR-372-5p
MSTRG.49059.1	RTL8B	has-miR-452-5p
MSTRG.10627.1	ADAMTS5	has-miR-373-3p
ENST00000659029	COL4A2	has-miR-224-3p
MSTRG.47563.1	STAC	has-miR-92b-3p
MSTRG.38525.1	SEMA6D	has-miR-582-5p
MSTRG.53441.1	CHIC1	has-miR-4427
MSTRG.9288.3	SCIN	has-miR-1303
MSTRG.39084.1	CDH13	has-miR-203a-3p
ENST00000666970	SGCE	has-miR-224-5p
MSTRG.51623.13	HECW2	has-miR-142-3p
MSTRG.40714.1	DSEL	has-miR-6720-5p
MSTRG.37033.3	INHBB	has-miR-509-5p
MSTRG.38611.1	SLC16A7	has-miR-1908-5p
ENST00000647388	LUM	has-miR-342-3p
MSTRG.28646.1		has-miR-450b-5p
		has-miR-934
		has-miR-302a-5p
		has-miR-3065-5p
		has-miR-424-5p
		has-miR-371a-5p
		has-miR-506-3p
		has-miR-34c-5p
		has-miR-142-5p
		has-miR-2682-5p
		has-miR-503-5p
		has-miR-582-3p
		has-miR-1247-5p
		has-miR-1247-3p
		has-miR-424-3p
		has-miR-372-3p
